# Epidemiological Forecasting with Model Reduction of Compartmental Models. Application to the COVID-19 Pandemic

**DOI:** 10.3390/biology10010022

**Published:** 2020-12-31

**Authors:** Athmane Bakhta, Thomas Boiveau, Yvon Maday, Olga Mula

**Affiliations:** 1 Service de Thermo-Hydraulique et de Mécanique des Fluides, CEA, Université Paris-Saclay, 91191 Gif-sur-Yvette, France; athmane.bakhta@cea.fr; 2 Institut Carnot Smiles, Sorbonne Université, 75005 Paris, France; thomas.boiveau@upmc.fr; 3 Sorbonne Université and Université de Paris, CNRS, Laboratoire Jacques-Louis Lions (LJLL), F-75005 Paris, France; maday@ann.jussieu.fr; 4 Institut Universitaire de France, 75005 Paris, France; 5 CEREMADE, CNRS, UMR 7534, Université Paris-Dauphine, PSL University, 75016 Paris, France; 6 Inria, Commedia Team, 75012 Paris, France

**Keywords:** COVID-19, epidemiology, forecasting, model reduction, reduced basis

## Abstract

**Simple Summary:**

Using tools from the reduced order modeling of parametric ODEs and PDEs, including a new positivity-preserving greedy reduced basis method, we present a novel forecasting method for predicting the propagation of an epidemic. The method takes a collection of highly detailed compartmental models (with different initial conditions, initial times, epidemiological parameters and numerous compartments) and learns a model with few compartments which best fits the available health data and which is used to provide the forecasts. We illustrate the promising potential of the approach to the spread of the current COVID-19 pandemic in the case of the Paris region during the period from March to November 2020, in which two epidemic waves took place.

**Abstract:**

We propose a forecasting method for predicting epidemiological health series on a two-week horizon at regional and interregional resolution. The approach is based on the model order reduction of parametric compartmental models and is designed to accommodate small amounts of sanitary data. The efficiency of the method is shown in the case of the prediction of the number of infected people and people removed from the collected data, either due to death or recovery, during the two pandemic waves of COVID-19 in France, which took place approximately between February and November 2020. Numerical results illustrate the promising potential of the approach.

## 1. Introduction

Providing reliable epidemiological forecasts during an ongoing pandemic is crucial to mitigate the potentially disastrous consequences for global public health and the economy. As the ongoing pandemic of COVID-19 sadly illustrates, this is a daunting task in the case of new diseases due to the incomplete knowledge of the behavior of the disease and the heterogeneities and uncertainties in the health data count. Despite these difficulties, many forecasting strategies exist, and we can cast them into two main categories: the first type is purely data-based and involves statistical and learning methods such as time series analysis, multivariate linear regression, grey forecasting or neural networks [1,2,3,4,5]; the second approach uses epidemiological models, which are appealing since they provide an interpretable insight of the mechanisms of the outbreak. They also provide high flexibility in the level of detail to describe the evolution of a pandemic, ranging from simple compartmental models that divide the population into a few exclusive categories to highly detailed descriptions involving numerous compartments or even agent-based models (see, e.g., [6,7,8] for general references on mathematical epidemiological models and [9,10,11] for some models focused on COVID-19). One salient drawback of using epidemiological models for forecasting purposes lies in the very high uncertainty in the estimation of the relevant parameters. This is due to the fact that the parameters cannot often be inferred from real observations, and the available data are insufficient or too noisy to provide any reliable estimation. The situation is aggravated by the fact that the number of parameters can quickly become large even in moderately simple compartmental models [10]. As a result, forecasting with these models involves making numerous a priori hypotheses which can sometimes be difficult to justify by data observations.

In this paper, our goal is to forecast the time-series of infected, removed and dead patients with compartmental models that involve as few parameters as possible in order to infer these series solely from the data. The available data are only given for hospitalized people; one can nevertheless estimate the total number of infected people through an adjustment factor taken from the literature. Such a factor takes into account the proportion of asymptomatic people and infected people who do not go to hospital. The model that includes the least number of parameters is probably the susceptible–infected–removed (SIR) model [12], which is based on a partition of the population into the following groups:

- Uninfected people, called susceptible (S);
- Infected and contagious people (I), with more or less marked symptoms;
- People removed (R) from the infectious process, either because they were cured or unfortunately died after being infected.

If *N* denotes the total population size that we assume to be constant over a certain time interval [0,T], we have

N=S(t)+I(t)+R(t),∀t∈[0,T],

and the evolution from *S* to *I* and from *I* to *R* is given for all t∈[0,T] by

$$\begin{array}{cc} \frac{dS}{dt}\left(t\right) & =-\frac{\beta I(t)S(t)}{N}\\ \frac{dI}{dt}\left(t\right) & =\frac{\beta I(t)S(t)}{N}-\gamma I\left(t\right)\\ \frac{dR}{dt}\left(t\right) & =\gamma I(t). \end{array}$$

The SIR model has only two parameters:

- γ>0 represents the recovery rate. In other words, its inverse $\gamma^{-1}$ can be interpreted as the length (in days) of the contagious period;
- β>0 is the transmission rate of the disease. It essentially depends on two factors: the contagiousness of the disease and the contact rate within the population. The larger this second parameter is, the faster the transition from susceptible to infectious will be. As a consequence, the number of hospitalized patients may increase very quickly and may lead to a collapse of the health system [13]. Strong distancing measures such as confinement can effectively act on this parameter [14,15], helping to keep it low.

Our forecasting strategy is motivated by the following observation: by allowing the parameters β and γ to be time-dependent, we can find optimal coefficients $\beta^{*}\left(t\right)$ and $\gamma^{*}\left(t\right)$ that exactly fit any series of infected and removed patients. In other words, we can perfectly fit any observed health series with an SIR model with time-dependent coefficients.

As we explain below, the high fitting power stems from the fact that the parameters β and γ are searched in $L^{\infty }\left(\left[0,T\right],R_{+}\right)$—the space of essentially bounded measurable functions. For our forecasting purposes, however, this space is too large to give any predictive power, and we need to find a smaller manifold that simultaneously has good fitting and forecasting properties. To this end, we developed a method based on model order reduction. The idea of the method was to find a space with a reduced dimension that can host the dynamics of the current epidemic. This reduced space is learnt from a series of detailed compartmental models based on precise underlying mechanisms of the disease. One major difficulty in these models is the fitting of the correct parameters. In our approach, we do not seek to estimate these parameters; instead, we consider a large range of possible parameter configurations with a uniform sampling that allows us to simulate virtual epidemic scenarios in a longer range than the fitting window [0,T]. We next cast each virtual epidemic from the family of detailed compartmental models into the family of SIR models with time-dependent coefficients, as explained below. This procedure yields time-dependent parameters β and γ for each detailed virtual epidemic. The set of all such β (or γ) is then condensed into a reduced basis with a small dimension. We finally use the available health data on the time window [0,T] to find the functions β and γ from the reduced space that best fit the current epidemic over [0,T]. Since the reduced basis functions are defined over a longer time range [0,T+τ] with τ>0 (e.g., two weeks), the strategy automatically provides forecasts from *T* to T+τ. Its accuracy will be related to the pertinence of the mechanistic mathematical models that have been used in the learning process.

We note that an important feature of our approach is that all virtual simulations are considered equally important in the first stage, and the procedure automatically learns what the best scenarios (or linear combinations of scenarios) to describe the available data are. Moreover, the approach even mixes different compartmental models to accommodate these available data. This is in contrast to other existing approaches which introduce a strong a priori belief regarding the quality of a certain particular model. Note also that we can add models related to other illness and use the large manifold to fit a possible new epidemic. It is also possible to mix the current approach with other purely statistical or learning strategies by means of expert aggregation. One salient difference with these approaches which is important to emphasize is that our method hinges on highly detailed compartmental models which have clear epidemiological interpretations. Our collapsing methodology into the time-dependent SIR is a way of “summarizing” the dynamics into a few terms. One may expect that reducing the number of parameters comes at the cost of losing the interpretability of parameters, and this is true in general. Nevertheless, the numerical results of the present work show that a reasonable tradeoff between the “reduction of the number of parameters” and “interpretability of these few parameters” can be achieved.

The paper is organized as follows. In Section 2, we present the forecasting method in the case of a single region with a constant population. For this, in Section 2.1, we briefly introduce the epidemiological models involved in the procedure, namely the SIR model with time-dependent coefficients and more detailed compartmental models used for the training step. In Section 2.2, after proving that the SIR model with time-dependent coefficients in $L^{\infty }\left(\left[0,T\right]\right)$ is able to fit any admissible epidemiological evolution (as explained below), we present the main steps of the forecasting method. The method involves a collapsing step from detailed models to SIR models with time-dependent coefficients and model reduction techniques. We detail these points in Section 2.3 and Section 2.4. In Section 3, we explain how the method can easily be extended to a multi-regional context involving population mobility and regional health data observations (provided, of course, that mobility data are available). In Section 3.1, we begin by clarifying that the nature of the mobility data will dictate the kind of multi-regional SIR model to use in this context. In Section 3.2, we outline how to adapt the main steps of the method to the multi-regional case. Finally, in Section 4, we present numerical results for the the two pandemic waves of COVID-19 in France in 2020, which took place approximately between February and November 2020. Concluding comments are given in Section 5, followed by two Appendix A and Appendix B that present details about the processing of the data noise and the forecasting error.

## 2. Methodology for a Single Region

For the sake of clarity, we first consider the case of a single region with a constant population and no population fluxes with other regions. Here, the term region is generic and may be applied to very different geographical scales, ranging from a full country to a department within a country or even smaller partitions of a territory.

### 2.1. Compartmental Models

The final output of our method is a mono-regional SIR model with time-dependent coefficients as explained below. This SIR model with time-dependent coefficients is evaluated with reduced modeling techniques involving a large family of models with finer compartments proposed in the literature. Before presenting the method in the next section, we here introduce all the models that we use in this paper along with useful notations for the rest of the paper.

#### 2.1.1. SIR Models with Time-Dependent Parameters

We fit and forecast the series of infected and removed patients (dead and recovered) with SIR models where the coefficients β and γ are time-dependent:

$$\begin{array}{cc} \frac{dS}{dt}\left(t\right) & =-\frac{\beta (t)I(t)S(t)}{N}\\ \frac{dI}{dt}\left(t\right) & =\frac{\beta (t)I(t)S(t)}{N}-\gamma \left(t\right)I\left(t\right)\\ \frac{dR}{dt}\left(t\right) & =\gamma (t)I(t). \end{array}$$

In the following, we use bold-faced letters for past-time quantities. For example, f:={f(t):0≤t≤T} for any function $f\in L^{\infty }\left(\left[0,T\right]\right)$. Using this notation, for any given β and $\gamma \in L^{\infty }\left(\left[0,T\right]\right)$ we denote by

(S,I,R)=SIR(β,γ,[0,T])

the solution of the associated SIR dynamics in [0,T].

#### 2.1.2. Detailed Compartmental Models

Models involving many compartments offer a detailed description of epidemiological mechanisms at the expense of involving many parameters. In our approach, we use them to generate virtual scenarios. One of the initial motivations behind the present work is to provide forecasts for the COVID-19 pandemic; thus, we have selected the two following models which are specific for this disease, but note that any other compartmental model [9,10,16] or agent-based simulation could also be used.

**Figure 1:**
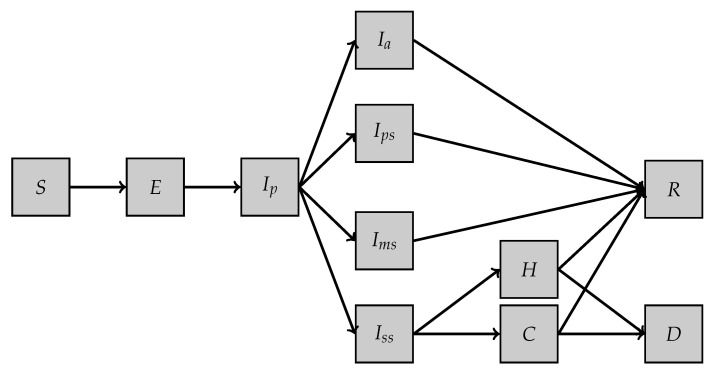
SEI5CHRD model.

- First model, SEI5CHRD: This model is inspired by the one proposed in [10]. It involves 11 different compartments and a set of 19 parameters (see Table 1). The dynamics of the model are illustrated in Figure 1, and the system of equations reads as follows:

$$\begin{array}{cc} \frac{dS}{dt}\left(t\right) & =-\frac{1}{N}S\left(t\right)\left(\beta_{p}I_{p}\left(t\right)+\beta_{a}I_{a}\left(t\right)+\beta_{ps}I_{ps}\left(t\right)+\beta_{ms}I_{ms}\left(t\right)+\beta_{ss}I_{ss}\left(t\right)+\beta_{H}H\left(t\right)+\beta_{C}C\left(t\right)\right)\\ \frac{dE}{dt}\left(t\right) & =\frac{1}{N}S\left(t\right)\left(\beta_{p}I_{p}\left(t\right)+\beta_{a}I_{a}\left(t\right)+\beta_{ps}I_{ps}\left(t\right)+\beta_{ms}I_{ms}\left(t\right)+\beta_{ss}I_{ss}\left(t\right)+\beta_{H}H\left(t\right)+\beta_{C}C\left(t\right)\right)-\varepsilon E\left(t\right)\\ \frac{dI_{p}}{dt}\left(t\right) & =\varepsilon E\left(t\right)-\mu_{p}I_{p}\left(t\right)\\ \frac{dI_{a}}{dt}\left(t\right) & =p_{a}\mu_{p}I_{p}\left(t\right)-\mu I_{a}\left(t\right)\\ \frac{dI_{ps}}{dt}\left(t\right) & =p_{ps}\left(1-p_{a}\right)\mu_{p}I_{p}\left(t\right)-\mu I_{ps}\left(t\right)\\ \frac{dI_{ms}}{dt}\left(t\right) & =p_{ms}\left(1-p_{a}\right)\mu_{p}I_{p}\left(t\right)-\mu I_{ms}\left(t\right)\\ \frac{dI_{ss}}{dt}\left(t\right) & =p_{ss}\left(1-p_{a}\right)\mu_{p}I_{p}\left(t\right)-\mu I_{ss}\left(t\right)\\ \frac{dC}{dt}\left(t\right) & =p_{c}\mu I_{ss}\left(t\right)-\left(\lambda_{C,R}+\lambda_{C,D}\right)C\left(t\right)\\ \frac{dH}{dt}\left(t\right) & =\left(1-p_{c}\right)\mu I_{ss}\left(t\right)-\left(\lambda_{H,R}+\lambda_{H,D}\right)H\left(t\right)\\ \frac{dR}{dt}\left(t\right) & =\lambda_{C,R}C\left(t\right)+\lambda_{H,R}H\left(t\right)\\ \frac{dD}{dt}\left(t\right) & =\lambda_{C,D}C\left(t\right)+\lambda_{H,D}H\left(t\right) \end{array}$$

The different parameters involved in the model are described in Table 2 and detailed in the appendix of [10].

We denote by

$$\begin{array}{cc} u=\mathrm{SEI}5\mathrm{CHRD}(u_{0},\beta_{p}, & \beta_{a},\beta_{ps},\beta_{ms},\beta_{ss},\beta_{H},\beta_{C},\\ & \varepsilon ,\mu_{p},p_{a},\mu ,p_{ps},p_{ms},p_{ss},p_{C},\\ & \lambda_{CR},\lambda_{CD},\lambda_{HR},\lambda_{HD},\left[0,T\right]) \end{array}$$

the parameter-to-solution map where $u=(S,E,I_{p},I_{a},I_{\mathrm{ps}},I_{\mathrm{ms}},I_{\mathrm{ss}},C,H,R,D)$.

**Figure 2:**
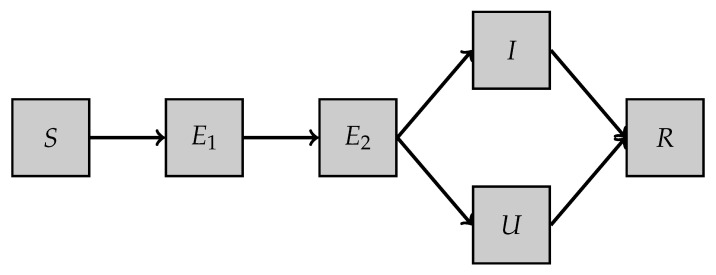
SE2IUR model.

- Second model, SE2IUR: This model is a variant of the one proposed in [9]. It involves five different compartments (see Table 3) and a set of six parameters. The dynamics of the model are illustrated in Figure 2 and the set of equations is as follows:
  $$\begin{array}{cc} \frac{dS}{dt}\left(t\right) & =-\frac{1}{N}\beta S\left(t\right)\left(E_{2}\left(t\right)+U\left(t\right)+I\left(t\right)\right)\\ \frac{dE_{1}}{dt}\left(t\right) & =\frac{1}{N}\beta S\left(t\right)\left(E_{2}\left(t\right)+U\left(t\right)+I\left(t\right)\right)-\delta E_{1}\left(t\right)\\ \frac{dE_{2}}{dt}\left(t\right) & =\delta E_{1}\left(t\right)-\sigma E_{2}\left(t\right)\\ \frac{dI}{dt}\left(t\right) & =\nu \sigma E_{2}\left(t\right)-\gamma_{1}I\left(t\right)\\ \frac{dU}{dt}\left(t\right) & =\left(1-\nu \right)\sigma E_{2}\left(t\right)-\gamma_{2}U\left(t\right)\\ \frac{dR}{dt}\left(t\right) & =\gamma_{1}I\left(t\right)+\gamma_{2}U\left(t\right) \end{array}$$
  We denote by
  $$u=\mathrm{SE}2\mathrm{IUR}(u_{0},\beta ,\delta ,\sigma ,\nu ,\gamma_{1},\gamma_{2},\left[0,T\right])$$
  the parameter-to-solution map where u=(S,E1,E2,I,U,R). The different parameters involved in the model are described in Table 4.

- Generalization: In the following, we abstract the procedure as follows. For any Detailed_Model with *d* compartments involving a vector $\mu \in R^{p}$ of *p* parameters, we denote by
  $$u\left(\mu \right)=\mathrm{Detailed}\_\mathrm{Model}\left(\mu ,\left[0,\tilde{T}\right]\right),\;u\left(\mu \right)\in L^{\infty }\left(\left[0,\tilde{T}\right],R^{d}\right)$$
  the parameter-to-solution map, where $\tilde{T}$ is some given time simulation that can be as large as desired because this is a virtual scenario. Note that, because the initial condition of the illness at time 0 is not known, we include the initial condition $u_{0}$ in the parameter set.

### 2.2. Forecasting Based on Model Reduction of Detailed Models

We assume that we are given health data in a time window [0,T], where T>0 is assumed to be the present time. The observed data are the series of infected people, denoted $I_{\mathrm{obs}}$, and removed people, denoted $R_{\mathrm{obs}}$. They are usually given at a national or a regional scale and on a daily basis. For our discussion, it is useful to work with time-continuous functions, and $t\to I_{\mathrm{obs}}\left(t\right)$ denotes the piecewise constant approximation in [0,T] from the given data (and similarly for $R_{\mathrm{obs}}\left(t\right)$). Our goal is to give short-term forecasts of the series in a time window τ>0 whose size is about two weeks. We denote by I(t) and R(t) the approximations to the series $I_{\mathrm{obs}}\left(t\right)$ and $R_{\mathrm{obs}}\left(t\right)$ at any time t∈[0,T].

As mentioned above, we propose to fit the data and provide forecasts with SIR models with time-dependent parameters β and γ. The main motivation for using such a simple family is that it possesses optimal fitting and forecasting properties for our purposes, as explained above. We define the cost function

(1) $$J(\beta ,\gamma \;|\;I_{\mathrm{obs}}\left(t\right),R_{\mathrm{obs}}\left(t\right),\left[0,T\right]):=\int_{0}^{T}\left(|I\left(t\right)-I_{\mathrm{obs}}{\left(t\right)|}^{2}+{\left|R\left(t\right)-R_{\mathrm{obs}}\left(t\right)\right|}^{2}\right)dt$$

such that

(S,I,R)=SIR(β,γ,[0,T]),

and the fitting problem can be expressed at the continuous level as the optimal control problem of finding

(2) $$J^{*}=\underset{\left(\beta ,\gamma \right)\in L^{\infty }\left(\left[0,T\right]\right)\times L^{\infty }\left(\left[0,T\right]\right)}{\inf}J\left(\beta ,\gamma \;|\;I_{\mathrm{obs}},R_{\mathrm{obs}},\left[0,T\right]\right).$$

The following result ensures the existence of a unique minimizer under very mild constraints.

**Proposition** **1.** *Let*$N\in N^{*}$andT>0. *For any real-valued functions*$S_{obs},I_{obs},R_{obs}$*of class*$C^{1}$, *defined on*[0,T]*satisfying*

*(i)*  $S_{obs}\left(t\right)+I_{obs}\left(t\right)+R_{obs}\left(t\right)=N$*for every*t∈[0,T],
*(ii)*  $S_{obs}$*in nonincreasing on*[0,T],
*(iii)*  $R_{obs}$*is nondeacreasing on*[0,T],

*there exists a unique minimizer*$\left(\beta_{obs}^{*},\gamma_{obs}^{*}\right)$*to Equation* (2).

**Proof.**  One can set

(3) $$\begin{array}{c} \left\{\begin{array}{cc} \beta_{\mathrm{obs}}^{*}\left(t\right) & :=-\frac{N}{I_{\mathrm{obs}}\left(t\right)S_{\mathrm{obs}}\left(t\right)}\frac{dS_{\mathrm{obs}}}{dt}\left(t\right)\\ \gamma_{\mathrm{obs}}^{*}\left(t\right) & :=\frac{1}{I_{\mathrm{obs}}\left(t\right)}\frac{dR_{\mathrm{obs}}}{dt}\left(t\right) \end{array}\right. \end{array}$$

so that

$$\left(S_{\mathrm{obs}},I_{\mathrm{obs}},R_{\mathrm{obs}}\right)=\mathrm{SIR}\left(\beta^{*},\gamma^{*},\left[0,T\right]\right)$$

and

$$J(\beta_{\mathrm{obs}}^{*},\gamma_{\mathrm{obs}}^{*},\left[0,T\right])=0$$

which obviously implies that $J^{*}=0$.    □

Note that one can equivalently set

$$\begin{array}{c} \left\{\begin{array}{cc} \beta_{\mathrm{obs}}^{*}\left(t\right) & :=-\frac{N}{I_{\mathrm{obs}}\left(t\right)S_{\mathrm{obs}}\left(t\right)}\frac{dS_{\mathrm{obs}}}{dt}\left(t\right)\\ \gamma_{\mathrm{obs}}^{*}\left(t\right) & :=\frac{1}{I_{\mathrm{obs}}\left(t\right)}\left[\frac{dI_{\mathrm{obs}}}{dt}\left(t\right)-\frac{\beta_{\mathrm{obs}}^{*}\left(t\right)I_{\mathrm{obs}}\left(t\right)S_{\mathrm{obs}}\left(t\right)}{N}\right] \end{array}\right. \end{array}$$

or again

$$\begin{array}{c} \left\{\begin{array}{cc} \gamma_{\mathrm{obs}}^{*}\left(t\right) & :=\frac{1}{I_{\mathrm{obs}}\left(t\right)}\frac{dR_{\mathrm{obs}}}{dt}\left(t\right)\\ \beta_{\mathrm{obs}}^{*}\left(t\right) & :=\frac{N}{I_{\mathrm{obs}}\left(t\right)S_{\mathrm{obs}}\left(t\right)}\left[\frac{dI_{\mathrm{obs}}}{dt}\left(t\right)-\gamma_{\mathrm{obs}}^{*}\left(t\right)I_{\mathrm{obs}}\left(t\right)\right] \end{array}\right. \end{array}$$

This simple observation means that there exists a time-dependent SIR model which can perfectly fit the data of any (epidemiological) evolution that satisfies properties (i), (ii), and (iii). In particular, we can perfectly fit the series of sick people with a time-dependent SIR model (with a smoothing of the local oscillations due to noise). Since the health data are usually given on a daily basis, we can approximate $\beta_{\mathrm{obs}}^{*},\gamma_{\mathrm{obs}}^{*}$ by approximating the derivatives by classical finite differences in Equation (3).

The fact that we can build $\beta_{\mathrm{obs}}^{*}$ and $\gamma_{\mathrm{obs}}^{*}$ such that $J\left(\beta_{\mathrm{obs}}^{*},\gamma_{\mathrm{obs}}^{*}\right)=J^{*}=0$ implies that the family of time-dependent SIR models is rich enough not only to fit the evolution of any epidemiological series but also to deliver perfect predictions of the health data. It is however important to note that since the $\beta_{\mathrm{obs}}^{*},\gamma_{\mathrm{obs}}^{*}$ are derived exclusively from the data and depend on time, we lose the direct interpretations of these coefficients in terms of the length of the contagious period or the transmission rate that these coefficients present when they are considered constant. The great approximation power comes also at the cost of defining the parameters β and γ in $L^{\infty }\left(\left[0,T\right]\right)$ which is a space that is too large to be able to define any feasible prediction strategy.

In order to pin down a smaller manifold where these parameters may vary without sacrificing much in terms of the fitting and forecasting power, our strategy is the following:

1. Learning phase: The fundamental hypothesis of our approach is that the specialists of epidemiology have understood the mechanisms of infection transmission sufficiently well. The second hypothesis is that these accurate models suffer from the proper determination of the parameters they contain. We thus propose to generate a large number of virtual epidemics, following these mechanistic models, with parameters that can be chosen in the vicinity of the suggested parameter values, including the different initial conditions.
  (a) Generate virtual scenarios using detailed models with constant coefficients:
    - Define the notion of a Detailed_Model which is most appropriate for the epidemiological study. Several models could be considered. For our numerical application, the detailed models are defined in Section 2.1.
    - Define an interval range $P\subset R^{p}$ where the parameters μ of etailed_Model vary. We call the solution manifold U the set of virtual dynamics over [0,T+τ], namely
      U:={u(μ)=Detailed_Model(μ,[0,T+τ]):μ∈P}.
    - Draw a finite training set
      $$P_{\mathrm{tr}}=\left\{\mu_{1},\ldots ,\mu_{K}\right\}\subseteq P$$
      of K≫1 parameter instances and compute u(μ)=Detailed_Model(μ,[0,T+τ]) for $\mu \in P_{\mathrm{tr}}$. Each u(μ) is a virtual epidemiological scenario. An important detail for our prediction purposes is that the simulations are done in [0,T+τ]; that is, we simulate not only in the fitting time interval but also in the prediction time interval. We call
      $$U_{\mathrm{tr}}=\left\{u\left(\mu \right)\;:\;\mu \in P_{\mathrm{tr}}\right\}$$
      the training set of all virtual scenarios.
  (b) Collapse every detailed model $u\left(\mu \right)\in U_{\mathrm{tr}}$ into an SIR model following the ideas explained in Section 2.3. For every u(μ), the procedure gives time-dependent parameters β(μ) and γ(μ) and associated SIR solutions (S,I,R)(μ) in [0,T+τ]. This yields the sets
    (4) $$B_{\mathrm{tr}}:=\left\{\beta \left(\mu \right)\;:\;\mu \in P_{\mathrm{tr}}\right\}\;\mathrm{and}\;G_{\mathrm{tr}}\left\{\gamma \left(\mu \right)\;:\;\mu \in P_{\mathrm{tr}}\right\}.$$
  (c) **Compute reduced models:**
    We apply model reduction techniques using $B_{\mathrm{tr}}$ and $G_{\mathrm{tr}}$ as training sets in order to build two bases
    $$B_{n}=\mathrm{span}\left\{b_{1},\ldots ,b_{n}\right\},\;G_{n}=\mathrm{span}\left\{g_{1},\ldots ,g_{n}\right\}\subset L^{\ldots }\left(\left[0,T+\tau \right],R\right),$$
    which are defined over [0,T+τ]. The space $B_{n}$ is such that it approximates all functions $\beta \left(\mu \right)\in B_{\mathrm{tr}}$ well (or all $\gamma \left(\mu \right)\in G_{\mathrm{tr}}$ can be well approximated by elements of $G_{n}$). In Section 2.4, we outline the methods we have explored in our numerical tests.
2. Fitting on the reduced spaces: We next solve the fitting problem (2) in the interval [0,T] by searching β (or γ) in $B_{n}$ (or in $G_{n}$) instead of in $L^{\infty }\left(\left[0,T\right]\right)$; that is,
  (5) $$J_{\left(B_{n},G_{n}\right)}^{*}=\min_{\left(\beta ,\gamma \right)\in B_{n}\times G_{n}}J\left(\beta ,\gamma \;|\;I_{\mathrm{obs}},R_{\mathrm{obs}},\left[0,T\right]\right).$$
  Note that the respective dimensions of $B_{n}$ and $G_{n}$ can be different; for simplicity, we consider them to be equal in the following. Obviously, since $B_{n}$ and $G_{n}\subset L^{\infty }\left(\left[0,T\right]\right)$, we obtain
  $$J^{*}\leq J_{\left(B_{n},G_{n}\right)}^{*},$$
  but we numerically observe that the function $n\mapsto J_{\left(B_{n},G_{n}\right)}^{*}$ decreases very rapidly as *n* increases, which indirectly confirms the fact that the manifold generated by the two above models accommodates the COVID-19 epidemic well.
  The solution of problem (5) gives us the coefficients ${\left(c_{i}^{*}\right)}_{i=1}^{n}$ and ${\left({\tilde{c}}_{i}^{*}\right)}_{i=1}^{n}\in R^{n}$ such that the time-dependent parameters
  $$\begin{array}{cc} \beta_{n}^{*}\left(t\right) & =\sum_{i=1}^{n}c_{i}^{*}b_{i}\left(t\right),\;\forall t\in \left[0,T+\tau \right],\\ \gamma_{n}^{*}\left(t\right) & =\sum_{i=1}^{n}{\tilde{c}}_{i}^{*}g_{i}\left(t\right). \end{array}$$
  achieve the minimum (5).
3. Forecast: For a given dimension *n* of the reduced spaces, we propagate in [0,T+τ] the associated SIR model, as follows:
  $$\left(S_{n}^{*},I_{n}^{*},R_{n}^{*}\right)=\mathrm{SIR}\left(\beta_{n}^{*},\gamma_{n}^{*},\left[0,T+\tau \right]\right)$$
  The values $I_{n}^{*}\left(t\right)$ and $R_{n}^{*}\left(t\right)$ for t∈[0,T[ are by construction close to the observed data $I_{\mathrm{obs}},R_{\mathrm{obs}}$ (up to some numerical optimization error). The values $I_{n}^{*}\left(t\right)$ and $R_{n}^{*}\left(t\right)$ for t∈[T,T+τ] are then used for prediction.
4. Forecast combination/aggregation of experts (optional step): By varying the dimension *n* and using different model reduction approaches, we can easily produce a collection of different forecasts, and thus the question of how to select the best predictive model arises. Alternatively, we can also resort to forecast combination techniques [17]: denoting $\left(I_{1},R_{1}\right),\ldots ,\left(I_{P},R_{P}\right)$ as the different forecasts, the idea is to search for an appropriate linear combination
  $$I^{\mathrm{FC}}\left(t\right)=\sum_{p=1}^{P}w_{p}I_{p}\left(t\right)$$
  and perform a similar operation for *R*. Note that these combinations do not need to involve forecasts from our methodology only; other approaches such as time series forecasts could also be included. One simple forecast combination is the average in which all alternative forecasts are given the same weight $w_{p}=1/P,\;p=1,\ldots ,P$. More elaborate approaches consist in estimating the weights that minimize a loss function involving the forecast error.

Before going into detail regarding some of the steps, three points should be highlighted:

1. To bring out the essential mechanisms, we have idealized some elements in the above discussion by omitting certain unavoidable discretization aspects. To start with, the ODE solutions cannot be computed exactly but only up to a certain level of accuracy given by a numerical integration scheme. In addition, the optimal control problems (2) and (5) are non-convex. As a result, in practice, we can only find a local minimum. Note, however, that modern solvers find solutions which are very satisfactory for all practical purposes. In addition, note that solving the control problem in a reduced space as in (5) could be interpreted as introducing a regularizing effect with respect to the control problem (2) in the full $L^{\infty }\left(\left[0,T\right]\right)$ space. It is to be expected that the search of global minimizers is facilitated in the reduced landscape.
2. routine-IR and routine-βγ: A variant for the fitting problem (5) as studied in our numerical experiments is to replace the cost function $J(\beta ,\gamma \;|\;I_{\mathrm{obs}},R_{\mathrm{obs}},\left[0,T\right])$ by the cost function
  (6) $$\tilde{J}\left(\beta ,\gamma \;|\;\beta_{\mathrm{obs}}^{*},\gamma_{\mathrm{obs}}^{*},\left[0,T\right]\right):=\int_{0}^{T}\left(|\beta -\beta_{\mathrm{obs}}^{*}{|}^{2}+|\gamma -\gamma_{\mathrm{obs}}^{*}{)|}^{2}\right)dt.$$
  In other words, we use the variables $\beta_{\mathrm{obs}}^{*}$ and $\gamma_{\mathrm{obs}}^{*}$ from (3) as observed data instead of working with the observed health series $I_{\mathrm{obs}}$, $R_{\mathrm{obs}}$. In Section 4, we refer to the standard fitting method as routine-IR and to this variant as routine-βγ.
3. The fitting procedure works both on the components of the reduced basis and the initial time of the epidemics to minimize the loss function; however, for simplicity, this last optimization is not reported here.

### 2.3. Details on Step 1-(b): Collapsing the Detailed Models into SIR Dynamics

Let

$$u\left(\mu \right)=\mathrm{Detailed}\_\mathrm{Model}\left(\mu ,\left[0,T+\tau \right]\right)\;\in L^{\infty }\left(\left[0,T+\tau \right],R^{d}\right)$$

be the solution in [0,T+τ] to a detailed model for a given vector of parameters $\mu \;\in \;R^{d}$. Here, *d* is possibly large (d=11 in the case of the SEI5CHRD model and d=5 in the case of SE2IUR’s model). The goal of this section is to explain how to collapse the detailed dynamics u(μ) into SIRdynamics with time-dependent parameters. The procedure can be understood as a projection of a detailed dynamics into the family of dynamics given by SIR models with time-dependent parameters.

For the SEI5CHRD model, we collapse the variables by setting

$$\begin{array}{cc} S^{\mathrm{col}} & =S+E\\ I^{\mathrm{col}} & =I_{p}+I_{a}+I_{\mathrm{ps}}+I_{\mathrm{ms}}+I_{\mathrm{ss}}+C+H\\ R^{\mathrm{col}} & =R+D \end{array}$$

Similarly, for the SE2IUR model, we set

$$\begin{array}{cc} S^{\mathrm{col}} & =S+E_{1i}\\ I^{\mathrm{col}} & =E_{2i}+I_{i}+U_{i}\\ R^{\mathrm{col}} & =R \end{array}$$

Note that $S^{\mathrm{col}}$, $I^{\mathrm{col}}$ and $R^{\mathrm{col}}$ depend on μ, but we have omitted this dependence to simplify the notation.

Once the collapsed variables are obtained, we identify the time-dependent parameters β and γ of the SIR model by solving the fitting problem

(7) $$\left(\beta ,\gamma \right)\in \underset{\left(\beta ,\gamma \right)\in L^{\infty }\left(\left[0,T+\tau \right],R\right)\times L^{\infty }\left(\left[0,T+\tau \right],R\right)}{arg inf}J\left(\beta ,\gamma \;|\;I^{\mathrm{col}},R^{\mathrm{col}},\left[0,T+\tau \right]\right)$$

where

(S,I,R)=SIR(β,γ,[0,T+τ]).

Note that problem (7) has the same structure as problem (2), with the difference arising from the fact that the collapsed variables $I^{\mathrm{col}}$, $R^{\mathrm{col}}$ in (7) play the role of the health data $I_{\mathrm{obs}}$, $R_{\mathrm{obs}}$ in (2). Therefore, it follows from Proposition 1 that problem (7) has a very simple solution as it suffices to apply formula (3) to solve it. Note here that the exact derivatives of $S^{\mathrm{col}}$, $I^{\mathrm{col}}$, and $R^{\mathrm{col}}$ can be obtained from the Detailed_Model.

Since the solution (β,γ) to (7) depends on the parameter μ of the detailed model, repeating the above procedure for every detailed scenario u(μ) for any $\mu \in P_{\mathrm{tr}}$ yields the two families of time-dependent functions $B_{\mathrm{tr}}=\left\{\beta \left(\mu \right)\;:\;\mu \in P_{\mathrm{tr}}\right\}$ and $G_{\mathrm{tr}}=\left\{\gamma \left(\mu \right)\;:\;\mu \in P_{\mathrm{tr}}\right\}$ defined in the interval [0,T+τ] as introduced in Section (4).

### 2.4. Details of Model Order Reduction

Model order reduction is a family of methods aiming at the approximation of a set of solutions of parametrized PDEs or ODEs (or related quantities) with linear spaces, which are called reduced models or reduced spaces. In our case, the sets to approximate are

B={β(μ):μ∈P}andG={γ(μ):μ∈P},

where each μ is the vector of parameters of the detailed model which take values over P, and β(μ) and γ(μ) are the associated time-dependent coefficients of the collapsed SIR evolution. In the following, we view B and G as subsets of $L^{2}\left(\left[0,T\right]\right)$, and we denote by ∥·∥ and $\langle \cdot ,\cdot \rangle$ its norm and inner product. Indeed, in view of Proposition 1, B and $G\subset L^{\infty }\left(\left[0,T\right]\right)\subset L^{2}\left(\left[0,T\right]\right)$.

Continuing the discussion if B (the same will hold for G), of we measure performance in terms of the worst error in the set B, the best possible performance that reduced models of dimension *n* can achieve is given by the Kolmogorov *n*-width:

$$d_{n}{\left(B\right)}_{L^{2}\left(\left[0,T\right]\right)}:=\underset{\begin{array}{c} Y\in L^{2}\left(\left[0,T\right]\right)\\ \dim(Y)\leq n \end{array}}{\inf}\max_{u\in B}∥u-P_{Y}u∥$$

where $P_{Y}$ is the orthogonal projection onto the subspace *Y*. In the case of measuring errors in an average sense, the benchmark is given by

$$\delta_{n}^{2}{\left(B,\nu \right)}_{L^{2}\left(\left[0,T\right]\right)}:=\underset{\begin{array}{c} Y\in L^{2}\left(\left[0,T\right]\right)\\ \dim(Y)=n \end{array}}{\inf}\int_{P}∥u\left(y\right)-P_{Y}{u\left(y\right)∥}^{2}d\nu \left(y\right)$$

where ν is some given measure on P.

In practice, building spaces that meet these benchmarks is generally not possible. However, it is possible to build sequences of spaces for which the error decay is comparable to that given by ${\left(d_{n}{\left(B\right)}_{L^{2}\left(\left[0,T\right]\right)}\right)}_{n}$ or ${\left(\delta_{n}{\left(B\right)}_{L^{2}\left(\left[0,T\right]\right)}\right)}_{n}$. As a result, when the Kolmogorov width decays quickly, the constructed reduced spaces will deliver a very good approximation of the set B with few modes (see [18,19,20,21]).

We next present the reduced models used in our numerical experiments. Other methods could, of course, be considered, and we refer readers to [22,23,24,25] for general references on model reduction. We continue the discussion in a fully discrete setting in order to simplify the presentation and keep it as close to the practical implementation as possible. All the claims below could be written in a fully continuous sense at the expense of introducing additional mathematical objects such as certain Hilbert–Schmidt operators to define the continuous version of Singular Value Decomposition (SVD).

We build the reduced models using the two discrete training sets of functions $B_{\mathrm{tr}}={\left\{\beta_{i}\right\}}_{i=1}^{K}$ and $G_{\mathrm{tr}}={\left\{\gamma_{i}\right\}}_{i=1}^{K}$ from B and G, where *K* denotes the number of virtual scenarios considered. The sets have been generated in step 1-(b) of our general pipeline (see Section 2.2).

We consider a discretization of the time interval [0,T+τ] into a set of $Q\in N^{*}$ points as follows: $\left\{t_{1}=0,\cdots ,t_{P}=T,\cdots ,t_{Q}=T+\tau \right\}$ where P<Q. Thus, we can represent each function $\beta_{i}$ as a vector of *Q* values

$$\beta_{i}={\left(\beta_{i}\left(t_{1}\right),\cdots ,\beta_{i}\left(t_{Q}\right)\right)}^{T}\in R_{+}^{Q}.$$

and thus assemble all the functions of the family ${\left\{\beta_{i}\right\}}_{i=1}^{K}$ into a matrix $M_{B}\in R_{+}^{Q\times K}$. The same remark applies for the family ${\left\{\gamma_{i}\right\}}_{i=1}^{K}$ which gives a matrix $M_{G}\in R_{+}^{Q\times K}$.

1. SVD: The eigenvalue decomposition of the correlation matrix $M_{B}^{T}M_{B}\in R^{K\times K}$ gives
  $$M_{B}^{T}M_{B}=V\Lambda V^{T},$$
  where $V=\left(v_{i,j}\right)\in R^{K\times K}$ is an orthogonal matrix and $\Lambda \in R^{K\times K}$ is a diagonal matrix with non-negative entries, which we denote as $\lambda_{i}$ and present in decreasing order. The $\ell^{2}\left(R^{Q}\right)$-orthogonal basis functions $\left\{b_{1},\ldots ,b_{K}\right\}$ are then given by the linear combinations
  $$b_{i}=\sum_{j=1}^{K}v_{j,i}\beta_{j},\;1\leq i\leq K.$$
  For n≤K, the space
  $$B_{n}=\mathrm{span}\left\{b_{1},\ldots b_{n}\right\}$$
  is the best *n*-dimensional space to approximate the set ${\left\{\beta_{i}\right\}}_{i=1}^{K}$ in the average sense. We have
  $$\delta_{n}{\left({\left\{\beta_{i}\right\}}_{i=1}^{K}\right)}_{\ell^{2}\left(R^{Q+1}\right)}={\left(\frac{1}{K}\sum_{i=1}^{K}∥\beta_{i}-P_{B_{n}}\beta_{i}{∥}_{\ell^{2}\left(R^{Q+1}\right)}^{2}\right)}^{1/2}={\left(\sum_{i>n}^{K}\lambda_{i}\right)}^{1/2}$$
  and the average approximation error is given by the sum of the tail of the eigenvalues.
  Therefore, the SVD method is particularly efficient if there is a fast decay of the eigenvalues, meaning that the set $B_{\mathrm{tr}}={\left\{\beta_{i}\right\}}_{i=1}^{K}$ can be approximated by only few modes. However, note that, by construction, this method does not ensure positivity in the sense that $P_{B_{n}}\beta_{i}\left(t\right)$ may become negative for some t∈[0,T] although the original function $\beta_{i}\left(t\right)\geq 0$ for all t∈[0,T]. This is due to the fact that the vectors $b_{i}$ are not necessarily nonnegative. As we will see later, in our study, ensuring positivity especially for extrapolation (i.e., forecasting) is particularly important and motivates the next methods.
2. Nonnegative Matrix Factorization (NMF, see [26,27]): NMF is a variant of SVD involving nonnegative modes and expansion coefficients. In this approach, we build a family of non-negative functions ${\left\{b_{i}\right\}}_{i=1}^{n}$ and we approximate each $\beta_{i}$ with a linear combination
  (8) $$\beta_{i}^{\mathrm{NMF}}=\sum_{j=1}^{n}a_{i,j}b_{j},\;1\leq i\leq K,$$
  where for every 1≤i≤K and 1≤j≤n, the coefficients $a_{i,j}\geq 0$ and the basis function $b_{j}\geq 0$. In other words, we solve the following constrained optimization problem:
  $$\left(W^{*},B^{*}\right)\in \underset{\left(W,B\right)\in R_{+}^{K\times n}\times R_{+}^{n\times Q}}{arg min}{∥M_{B}-WB∥}_{F}^{2}.$$
  We refer readers to [27] for further details on the NMF and its numerical aspects.
3. The Enlarged Nonnegative Greedy (ENG) algorithm with projection on an extended cone of positive functions: We now present our novel model reduction method, which is of interest in itself as it allows reduced models that preserve positivity and even other types of bounds to be built. The method stems from the observation that NMF approximates functions in the cone of positive functions of $\mathrm{span}{\left\{b_{i}\geq 0\right\}}_{i=1}^{n}$ since it imposes $a_{i,j}\geq 0$ in the linear combination (8). However, note that the positivity of the linear combination is not equivalent to the positivity of the coefficients $a_{i,j}$ since there are obviously linear combinations involving very small $a_{i,j}<0$ for some *j* which may still deliver a nonnegative linear combination $\sum_{j=1}^{n}a_{i,j}b_{j}$. We can thus widen the cone of linear combinations yielding positive values by carefully including these negative coefficients $a_{i,j}$. One possible strategy for this is proposed in Algorithm 1, which describes a routine that we call Enlarge_Cone. The routine
  $$\left\{\psi_{1},\ldots ,\psi_{n}\right\}=\mathrm{Enlarge}\_\mathrm{Cone}\left[\left\{b_{1},\ldots ,b_{n}\right\},\varepsilon \right]$$
  takes a set of nonnegative functions $\left\{b_{1},\ldots ,b_{n}\right\}$ as an input and modifies each function $b_{i}$ by iteratively adding negative linear combinations of the other basis functions $b_{j}$ for j≠i (see line 11 of the routine). The coefficients are chosen in an optimal way so as to maintain the positivity of the final linear combination while minimizing the $L^{\infty }$-norm. The algorithm returns a set of functions
  $$\psi_{i}=b_{i}-\sum_{j\neq i}\sigma_{j}^{i}b_{j},\;\forall i\in \left\{1,\ldots ,n\right\}$$
  with $\sigma_{j}^{i}\geq 0$. Note that the algorithm requires the setting of a tolerance parameter ε>0 for the computation of the $\sigma_{j}^{i}$.
  Once we have run Enlarge_Cone, the approximation of any function β is then sought as
  $$\beta^{\left(EC\right)}=\underset{c_{1},\ldots ,c_{n}\geq 0}{arg min}{∥\beta -\sum_{j=1}^{n}c_{j}\psi_{j}∥}_{L^{2}\left(\left[0,T+\tau \right]\right)}^{2}$$
  We emphasize that the routine is valid for any set of nonnegative input functions. We can thus apply Enlarge_Cone to the functions ${\left\{b_{i}\geq 0\right\}}_{i=1}^{n}$ from NMF but also to the functions selected by a greedy algorithm such as the following:
  - For n=1, find
    $$b_{1}=\underset{\beta \in {\left\{\beta_{i}\right\}}_{i=1}^{K}}{arg max}{∥\beta ∥}_{L^{2}\left(\left[0,T+\tau \right]\right)}^{2}$$
  - At step n>1, we have selected the set of functions $\left\{b_{1},\ldots ,b_{n-1}\right\}$. We next find
    $$b_{n}=\underset{\beta \in {\left\{\beta_{i}\right\}}_{i=1}^{K}}{arg max}\min_{c_{1},\ldots ,c_{n}\geq 0}{∥\beta -\sum_{j=1}^{n-1}c_{j}b_{j}∥}_{L^{2}\left(\left[0,T+\tau \right]\right)}^{2}$$
  In our numerical tests, we call the Enlarged Nonnegative Greedy (ENG) method the routine involving the above greedy algorithm combined with our routine Enlarge_Cone.
**Algorithm 1** Enlarge_Cone$\left[\left\{b_{1},\ldots ,b_{n}\right\},\varepsilon \right]\to \left\{\psi_{1},\ldots ,\psi_{n}\right\}$.**Input:** Set of nonnegative functions $\left\{b_{1},\ldots ,b_{n}\right\}$. Tolerance ε>0.  **for**
i∈{1,…,n}
**do**    Set $\sigma_{j}^{i}=0,\;\forall j\neq i.$    **for**
ℓ∈{1,…,n}
**do**      $\alpha_{\ell }^{i,*}=arg max\left\{\alpha \geq 0\;:\;\left[b_{i}-\sum_{j\neq i}\sigma_{j}^{i}b_{j}-\alpha b_{\ell }\right]\left(t\right)>0,\;\;\forall t\in \left[0,T+\tau \right]\right\}$      $\sigma_{\ell }^{i}=\sigma_{\ell }^{i}+\frac{\alpha_{\ell }^{i,*}}{2}$       **while**
$\alpha_{\ell }^{i,*}\geq \varepsilon$
**do**        $\alpha_{\ell }^{i,*}=arg max\left\{\alpha \geq 0\;:\;\left[b_{i}-\sum_{j\neq i}\sigma_{j}^{i}b_{j}-\alpha b_{\ell }\right]\left(t\right)>0,\;\;\forall t\in \left[0,T+\tau \right]\right\}$        $\sigma_{\ell }^{i}=\sigma_{\ell }^{i}+\frac{\alpha_{\ell }^{i,*}}{2}$       **end while**     **end for**    $\psi_{i}=b_{i}-\sum_{j\neq i}\sigma_{j}^{i}b_{j}$  **end for** **Output:**$\left\{\psi_{1},\ldots ,\psi_{n}\right\}$

  We remark that, if we work with positive functions that are upper bounded by a constant L>0, we can ensure that the approximations, denoted as Ψ, and written as a linear combination of basis functions, will also be between these bounds 0 and *L* by defining on the one hand, and as we have just done, a cone of positive functions generated by the above family ${\left\{\psi_{i}\right\}}_{i}$, and on the other hand, considering the base of the functions L−φ, φ to be above the set all greedy elements of the reduced basis, to which we also apply the enlargement of these positive functions. We then require the approximation to be written as a positive combination of the first (positive) functions and for L−Ψ to also be written as a combination with positive components in the second basis.
  In this frame, the approximation appears under the form of a least-square approximation with 2n linear constraints on the *n* coefficients, expressing the fact that the coefficients are nonnegative in the two above transformed bases.

In addition to the previous basis functions, it is possible to include more general/generic basis functions such as polynomial, radial, or wavelet functions in order to guarantee simple forecasting trends. For instance, one can add affine functions in order to include the possibility of predicting with a simple linear extrapolation to the range of possible forecasts offered by the reduced model. Given the overall exponential behavior of the health data, we have added an exponential function of the form $b_{0}\left(t\right)=\xi \exp\left(-\xi^{\prime }t\right)$ (or $g_{0}\left(t\right)=\psi \exp\left(-\psi^{\prime }t\right)$) to the reduced basis functions $\left\{b_{1},\ldots ,b_{n}\right\}$ (or $\left\{g_{1},\ldots ,g_{n}\right\}$) where the real-valued nonnegative parameters $\xi ,\xi^{\prime },\psi ,\psi^{\prime }$ are obtained through a standard exponential regression from $\beta_{\mathrm{obs}}^{*}$ (or $\gamma_{\mathrm{obs}}^{*}$) associated with the targeted profiles of infectious people; that is, the profiles defined in the time interval [0,T] that should be extrapolated to [*T*, τ]. In other words, the final reduced models that we use for forecasting are

$$B_{n+1}=\mathrm{span}\left\{b_{0},b_{1},\ldots ,b_{n}\right\},\;G_{n+1}=\mathrm{span}\left\{g_{0},g_{1},\ldots ,g_{n}\right\}\subset L^{\infty }\left(\left[0,T+\tau \right],R\right),$$

Indeed, including the exponential functions in the reduced models gives easy access to the overall behavior of the parameters β and γ; the rest of the basis functions generated from the training sets catch the higher-order approximations and allow then to improve the extrapolation.

**Remark** **1.** ***Reduced models on I={I(μ):μ∈P} and R={R(μ):μ∈P}*** *Instead of applying model reduction to the sets B and G, as we do in our approach, we could apply the above techniques directly to the sets of solutions I and R of the SIR models with time-dependent coefficients in B and G. In this case, the resulting approximation would however not follow SIR dynamics.*

## 3. Methodology for Multiple Regions Including Population Mobility Data

The forecasting method of Section 2.2 for a single region can be extended to the treatment of multiple regions involving population mobility. The prediction scheme is based on a multi-regional SIR with time-dependent coefficients. Compared to other more detailed models, the main advantage of our approach is that it drastically reduces the number of parameters to be estimated. Indeed, detailed multi-regional models such as multi-regional extensions of the above SEI5CHRD and SE2IUR models from Section 2.3 require a number of parameters that quickly grows with the number *P* of regions involved. Their calibration thus requires large amounts of data which, in addition, may be unknown, very uncertain, or not available. In a forthcoming paper, we will apply the fully multi-regional setting for the post-lockdown period.

The structure of this section is the same as above for the case of a single region. In Section 3.1, we begin by introducing the multi-regional SIR model with time-dependent coefficients and associated detailed models. As with any multi-regional model, mobility data are required as input data, and the nature and level of detail of the available data motivates certain choices regarding the modeling of the multi-regional SIR (as well as the other detailed models). We then present in Section 3.2 the general pipeline, in which we emphasize the high modularity of the approach.

### 3.1. Multi-Regional Compartmental Models

In the spirit of fluid flow modeling, there are essentially two ways of describing mobility between regions:

- In a Eulerian description, we take the regions as fixed references for which we record incoming and outgoing travels;
- In a Lagrangian description, we follow the motion of people living in a certain region and record their travels in the territory. We can expect this modeling to be more informative regarding the geographical spread of the disease, but it comes at the cost of additional details regarding the home region of the population.

Note that both descriptions hold at any coarse or fine geographical level, in the sense that what we call the regions could be taken to be full countries, departments within a country, or very small geographical partitions of a territory. We next describe the multi-regional SIR models with the Eulerian and Lagrangian descriptions of population fluxes, which form- the output of our methodology.

#### 3.1.1. Multi-Regional SIR Models with Time-Dependent Parameters

Eulerian description of population flux: Assume that we have *P* regions and the number of people in region *i* is $N_{i}$ for i=1,…,P. Due to mobility, the population in each region varies, so $N_{i}$ depends on *t*. However, the total population is assumed to be constant and equal to *N*; that is,

$$N=\sum_{i=1}^{P}N_{i}\left(t\right),\;\forall t\geq 0.$$

For any t≥0, let $\lambda_{i\to j}\left(t\right)\in \left[0,1\right]$ be the probability that people from *i* travel to *j* at time *t*. In other words, $\lambda_{i\to j}\left(t\right)N_{i}\left(t\right)\delta t$ is the number of people from region *i* that have travelled to region *j* between time *t* and t+δt. Note that we have

$$\sum_{j=1}^{P}\lambda_{i\to j}\left(t\right)=1,\;\forall t\geq 0.$$

Since, for any δt≥0,

$$N_{i}\left(t+\delta t\right)=N_{i}\left(t\right)-\sum_{j\neq i}\lambda_{i\to j}\left(t\right)N_{i}\left(t\right)\delta t+\sum_{j\neq i}\lambda_{j\to i}\left(t\right)N_{j}\left(t\right)\delta t$$

dividing by δt and taking the limit δt→0 yields

$$\frac{dN_{i}}{dt}\left(t\right)=-\sum_{j\neq i}\lambda_{i\to j}\left(t\right)N_{i}\left(t\right)+\sum_{j\neq i}\lambda_{j\to i}\left(t\right)N_{j}\left(t\right).$$

Note that we have

$$\sum_{i=1}^{P}\frac{dN_{i}}{dt}\left(t\right)=0,\;\forall t\geq 0.$$

Thus, $\sum_{i}N_{i}\left(t\right)=\sum_{i}N_{i}\left(0\right)=N$, which is consistent with our assumption that the total population is constant.

The time evolution of the $N_{i}$ is known in this case if we are given the $\lambda_{i\to j}\left(t\right)$ from Eulerian mobility data. In addition to these mobility data, we also have the data of the evolution of infected and removed people, and our goal is to fit a multi-regional SIR model that is in accordance with this data. Thus, we propose the following model.

Denoting $S_{i}$, $I_{i}$ and $R_{i}$ as the number of susceptible, infectious and removed people in region *i* at time *t*, we first have the relation

$$N_{i}\left(t\right)=S_{i}\left(t\right)+I_{i}\left(t\right)+R_{i}\left(t\right)\;\Leftrightarrow \;1=\frac{S_{i}\left(t\right)}{N_{i}\left(t\right)}+\frac{I_{i}\left(t\right)}{N_{i}\left(t\right)}+\frac{R_{i}\left(t\right)}{N_{i}\left(t\right)}.$$

Note that from the second relation, it follows that

(9) $$0=\frac{d}{dt}\frac{S_{i}}{N_{i}}+\frac{d}{dt}\frac{I_{i}}{N_{i}}+\frac{d}{dt}\frac{R_{i}}{N_{i}}.$$

To model the evolution between compartments, one possibility is the following SIR model:

(10) $$\begin{array}{c} \frac{d}{dt}\frac{S_{i}}{N_{i}}=-\left(\beta_{i}\lambda_{i\to i}\frac{I_{i}}{N_{i}}+\sum_{j\neq i}\beta_{j}\lambda_{j\to i}\frac{I_{j}}{N_{j}}\right)\frac{S_{i}}{N_{i}}\\ \frac{d}{dt}\frac{I_{i}}{N_{i}}=-\frac{d}{dt}\frac{S_{i}}{N_{i}}-\gamma_{i}\frac{I_{i}}{N_{i}}\\ \frac{d}{dt}\frac{R_{i}}{N_{i}}=\gamma_{i}\frac{I_{i}}{N_{i}}, \end{array}$$

The parameters $\beta_{i}$, $\gamma_{i}$, $N_{i}$ depend on *t*, but we have omitted this dependence for ease of reading. Introducing the compartmental densities

$$s_{i}=\frac{S_{i}}{N_{i}},\;i_{i}=\frac{I_{i}}{N_{i}},\;r_{i}=\frac{R_{i}}{N_{i}},$$

the system equivalently reads

(11) $$\begin{array}{c} \frac{d}{dt}s_{i}=-\left(\beta_{i}\lambda_{i\to i}i_{i}+\sum_{j\neq i}\beta_{j}\lambda_{j\to i}i_{j}\right)s_{i}\\ \frac{d}{dt}i_{i}=-\frac{d}{dt}s_{i}-\gamma_{i}i_{i}\\ \frac{d}{dt}r_{i}=\gamma_{i}i_{i}, \end{array}$$

Before going further, some points should be highlighted:

- The model is consistent in the sense that it satisfies (9), and when P=1, we recover the traditional SIR model;
- Under lockdown measures, $\lambda_{i\to j}\approx \delta_{i,j}$ and the population $N_{i}\left(t\right)$ remains practically constant. As a result, the evolution of each region is decoupled from the others, and each region can be addressed with a mono-regional approach;
- The use of $\beta_{j}$ in Equation (11) is debatable. When people from region *j* arrive in region *i*, it may be reasonable to assume that the contact rate is $\beta_{i}$;
- The use of $\lambda_{j\to i}$ in Equation (11) is also very debatable. The probability $\lambda_{j\to i}$ was originally defined to account for the mobility of people from region *j* to region *i* without specifying the compartment. However, in Equation (11), we need the probability of mobility of infectious people from region *j* to region *i*, which we denote by $\mu_{j\to i}$ in the following. It seems reasonable to think that $\mu_{j\to i}$ may be smaller than $\lambda_{j\to i}$, because as soon as people become symptomatic and suspect an illness, they will probably not move. Two possible options would be as follows:
  - We could try to estimate $\mu_{j\to i}$. If symptoms arise, for example, 2 days after infection and if people recover in 15 days on average, then we could say that $\mu_{j\to i}=2/15\lambda_{j\to i}$.
  - As the above seems to be quite empirical, another option would be to use $\lambda_{j\to i}$ and absorb the uncertainty in the values of the $\beta_{j}$ that can be fitted.
- We choose not to add mobility in the *R* compartment as this does not modify the dynamics of the epidemic spread; only adjustments in the population sizes are needed.

Lagrangian description of population flux: We call the above description Eulerian because we have fixed the regions as a fixed reference. Another possibility is to follow the trajectories of inhabitants of each region, in the same spirit as when we follow the trajectories of fluid particles.

Let $S_{i}$, $I_{i}$, and $R_{i}$ now be the number of susceptible, infectious and removed people who are resident in region i,i=1,…,P. It is reasonable to assume that $S_{i}\left(t\right)+I_{i}\left(t\right)+R_{i}\left(t\right)$ is constant in time. However, not all the residents of region *i* may be in that region at time *t*. Let $\lambda_{j\to k}^{\left(i\right)}\left(t\right)$ be the probability that susceptible people resident in *i* travel from region *j* to region *k* at time *t*. With this notation, $\lambda_{i\to i}^{\left(i\right)}\left(t\right)$ is the probability that susceptible people resident at *i* remain in region *i* at time *t*. Similarly, let $\mu_{j\to k}^{\left(i\right)}\left(t\right)$ be the probability that infectious people resident in *i* travel from region *j* to *k* at time *t*. Thus, the total number of susceptible and infectious people that are in region *i* at time *t* is

$$\begin{array}{cc} S_{i}\left(t\right) & =\sum_{k=1}^{P}\sum_{j=1}^{P}\left(\lambda_{j\to i}^{\left(k\right)}\left(t\right)-\lambda_{i\to j}^{\left(k\right)}\left(t\right)\right)S_{k}\left(t\right) \end{array}$$

$$\begin{array}{cc} I_{i}\left(t\right) & =\sum_{k=1}^{P}\sum_{j=1}^{P}\left(\mu_{j\to i}^{\left(k\right)}\left(t\right)-\mu_{i\to j}^{\left(k\right)}\left(t\right)\right)S_{k}\left(t\right) \end{array}$$

We can thus write the evolution over $S_{i}$, $I_{i}$, $R_{i}$ as

(12) $$\begin{array}{cc} \frac{dS_{i}}{dt} & =-\sum_{j=1}^{P}\sum_{k=1}^{P}\beta_{k}\left(t\right)\lambda_{j\to k}^{\left(i\right)}\left(t\right)S_{i}\left(t\right)I_{k}\left(t\right)\\ \frac{dI_{i}}{dt} & =-\frac{dS_{i}}{dt}-\gamma_{i}\left(t\right)I_{i}\left(t\right)\\ \frac{dR_{i}}{dt} & =\gamma_{i}\left(t\right)I_{i}\left(t\right) \end{array}$$

Note that $S_{i}\left(t\right)+I_{i}\left(t\right)+R_{i}\left(t\right)$ is constant, which is consistent with the fact that, in our model,

$$\frac{d}{dt}\left(S_{i}+I_{i}+R_{i}\right)=0.$$

We emphasize that, to implement this model, the Lagrangian mobility data $\lambda_{j\to k}^{\left(i\right)}$ are required for all $\left(i,j,k\right)\in {\left\{1,\ldots ,P\right\}}^{3}$.

Notation: In the following, we gather the compartmental variables in vectors

$$\vec{S}:={\left(S\right)}_{i=1}^{P},\;\vec{I}:={\left(I\right)}_{i=1}^{P},\;\vec{R}:={\left(R\right)}_{i=1}^{P}$$

as well as the time-dependent coefficients

$$\vec{\beta }={\left(\beta \right)}_{i=1}^{P},\;\vec{\gamma }={\left(\gamma \right)}_{i=1}^{P}.$$

For any $\vec{\beta }$ and $\vec{\gamma }\in {\left(L^{\infty }\left(\left[0,T\right]\right)\right)}^{P}$, we denote by

$$\left(\vec{S},\vec{I},\vec{R}\right)=\mathrm{Multiregional}\_\mathrm{SIR}\left(\vec{S}\left(0\right),\vec{I}\left(0\right),\vec{R}\left(0\right),\vec{\beta },\vec{\gamma },\left[0,T\right]\right)$$

the output of any of the above multi-regional SIR models. For simplicity, in what follows, we omit the initial condition in the notation.

#### 3.1.2. Detailed Multi-Regional Models with Constant Coefficients

In the spirit of the multi-regional SIR, one can formulate detailed multi-regional versions of more detailed models such as those introduced in Section 2.1. We omit the details for the sake of brevity.

### 3.2. Forecasting for Multiple Regions with Population Mobility

Similar to the mono-regional case, we assume that we are given health data in [0,T] in all regions. The observed data in region *i* are the series of infected people, denoted as $I_{i}^{\mathrm{obs}}$, and recovered people, denoted as $R_{i}^{\mathrm{obs}}$. They are usually given at a national or a regional scale and on a daily basis.

We propose to fit the data and provide forecasts with SIR models with time-dependent parameters $\beta_{i}$ and $\gamma_{i}$ for each region *i*. As in the mono-regional case, we can prove that such a simple family possesses optimal fitting properties for our purposes. In the current case, the cost function reads

$$\begin{array}{cc} \; & J(\vec{\beta },\vec{\gamma }\;|\;{\vec{I}}_{\mathrm{obs}},{\vec{R}}_{\mathrm{obs}},\left[0,T\right]):=\sum_{i=1}^{P}\int_{0}^{T}\left(|I_{i}\left(t\right)-I_{i}^{\mathrm{obs}}{\left(t\right)|}^{2}+{\left|R_{i}\left(t\right)-R_{i}^{\mathrm{obs}}\left(t\right)\right|}^{2}\right)dt\\ \; & \;\mathrm{such}\;\mathrm{that}\;\left(\vec{S},\vec{I},\vec{R}\right)=\mathrm{Multiregional}\_\mathrm{SIR}\left(\vec{\beta },\vec{\gamma },\left[0,T\right]\right), \end{array}$$

and the fitting problem is the optimal control problem of finding

(13) $$J^{*}=\underset{\vec{\beta },\vec{\gamma }\in {\left(L^{\infty }\left(\left[0,T\right]\right)\right)}^{P}\times {\left(L^{\infty }\left(\left[0,T\right]\right)\right)}^{P}}{\inf}J(\vec{\beta },\vec{\gamma }\;|\;{\vec{I}}_{\mathrm{obs}},{\vec{R}}_{\mathrm{obs}},\left[0,T\right]).$$

The following proposition ensures the existence of a unique minimizer under certain conditions. To prove this, it is useful to remark that any of the above multi-regional SIR models (see (11) and (12)) can be written in the general form

$$\begin{array}{cc} \frac{d\vec{S}}{dt} & =M\left(\Lambda \left(t\right),\vec{S}\left(t\right),\vec{I}\left(t\right)\right)\vec{\beta }\\ \frac{d\vec{I}}{dt} & =-\frac{d\vec{S}}{dt}-\mathrm{diag}\left(I\left(t\right)\right)\;\vec{\gamma }\\ \frac{d\vec{R}}{dt} & =\mathrm{diag}\left(I\left(t\right)\right)\vec{\gamma }, \end{array}$$

where, by a slight change of notation, the vectors $\vec{S}$, $\vec{I}$ and $\vec{R}$ are the densities of population in the case of the Eulerian approach (see Equation (11)). They are classical population numbers in the case of the Lagrangian approach (see Equation (12)). diag(I(t)) is the P×P diagonal matrix with diagonal entries given by the vector I(t). $M(\Lambda \left(t\right),\vec{S}\left(t\right),\vec{I}\left(t\right))$ is a matrix of size P×P that depends on the vectors of susceptible and infectious people $\vec{S}\left(t\right)$, $\vec{I}\left(t\right)$ and on the mobility matrix Λ. In the case of the Eulerian description, $\Lambda \left(t\right)={\left(\lambda_{i,j}\left(t\right)\right)}_{1\leq i,j\leq P}$ and in the case of the Lagrangian approach Λ(t) is the P×P×P tensor $\Lambda \left(t\right)={\left(\lambda_{j,k}^{\left(i\right)}\left(t\right)\right)}_{1\leq i,j,k\leq P}$. For example, in the case of the Eulerian model (12), the matrix *M* reads

(14) $$M\left(\Lambda \left(t\right),\vec{S}\left(t\right),\vec{I}\left(t\right)\right)=-\mathrm{diag}\left(\vec{S}\left(t\right)\right)\Lambda^{T}\mathrm{diag}\left(\vec{I}\left(t\right)\right)=-{\left(S_{i}\lambda_{i\to j}I_{j}\right)}_{1\leq i,j\leq P}$$

**Proposition** **2.** If $M(\Lambda \left(t\right),\vec{S}\left(t\right),\vec{I}\left(t\right))$ is invertible for all t∈[0,T], then there exists a unique minimizer $\left({\vec{\beta }}^{*},{\vec{\gamma }}^{*}\right)$ to problem (13).

**Proof.**  Since we assume that $M(\Lambda \left(t\right),\vec{S}\left(t\right),\vec{I}\left(t\right))$ is invertible for every t∈[0,T], we can set

$$\begin{array}{c} \left\{\begin{array}{cc} {\vec{\beta }}^{*}\left(t\right) & :=M^{-1}\left(t\right)\frac{d\vec{S}}{dt}\\ {\vec{\gamma }}^{*}\left(t\right) & :={\mathrm{diag}}^{-1}\left(I\left(t\right)\right)\frac{d\vec{R}}{dt} \end{array}\right. \end{array}$$

or equivalently

$$\begin{array}{c} \left\{\begin{array}{cc} {\vec{\beta }}^{*}\left(t\right) & :=M^{-1}\left(t\right)\frac{d\vec{S}}{dt}\\ {\vec{\gamma }}^{*}\left(t\right) & :=-{\mathrm{diag}}^{-1}\left(I\left(t\right)\right)\left(\frac{d\vec{I}}{dt}+M\left(\Lambda \left(t\right),\vec{S}\left(t\right),\vec{I}\left(t\right)\right){\vec{\beta }}^{*}\right) \end{array}\right. \end{array}$$

so that

$$\left({\vec{S}}_{\mathrm{obs}},{\vec{I}}_{\mathrm{obs}},{\vec{R}}_{\mathrm{obs}}\right)=\mathrm{Multiregional}\_\mathrm{SIR}\left({\vec{\beta }}^{*},{\vec{\gamma }}^{*},\left[0,T\right]\right)$$

and

$$J({\vec{\beta }}^{*},{\vec{\gamma }}^{*}\;|\;{\vec{I}}_{\mathrm{obs}},{\vec{R}}_{\mathrm{obs}},\left[0,T\right])=0$$

which implies that $J^{*}=0$. □

Before continuing, let us comment on the invertibility of $M(\Lambda \left(t\right),\vec{S}\left(t\right),\vec{I}\left(t\right))$ which is necessary in Proposition 2. A sufficient condition to ensure this is if the matrix is diagonally dominant row-wise or column-wise. This yields certain conditions on the mobility matrix Λ(t) with respect to the values of $\vec{S}\left(t\right)$, $\vec{I}\left(t\right)$. For example, if *M* is defined as in Equation (14), the matrix is diagonally dominant in each row if for every 1≤i≤P,

$$\lambda_{i\to i}>\sum_{j\neq i}\lambda_{i\to j}\frac{I_{j}}{I_{i}}.$$

Similarly, if for every 1≤j≤P,

$$\lambda_{j\to j}>\sum_{i\neq j}\lambda_{i\to j}\frac{S_{i}}{S_{j}},$$

then the matrix is diagonally dominant for each column and guarantees invertibility. Note that any of the above conditions is satisfied in situations with little or no mobility where $\lambda_{i\to i}\approx \delta_{i,j}$.

Now that we have exactly defined the set-up for the multi-regional case, we can follow the same steps in Section 2.2 to derive forecasts involving model reduction for the time-dependent variables $\vec{\beta }$ and $\vec{\gamma }$.

## 4. Numerical Results

In this section, we apply our forecasting method to the ongoing COVID-19 pandemic, which spread in the year 2020 in France and started approximately in February. Particular emphasis is placed on the first pandemic wave, for which we consider the period from 19 March to 20 May 2020. Due to the lockdown imposed between 17 March and 11 May, inter-regional population mobility was drastically reduced in that period. Studies using anonymized Facebook data have estimated the reduction to be 80% (see [28]). As a result, it is reasonable to treat each region independently from the rest, and we apply the mono-regional setting in Section 2. Here, we focus on the case of the Paris region, and we report different forecasting errors obtained using the methods described in Section 2. Some forecasts are also shown for the second wave for the Paris region between 24 September and 25 November.

The numerical results are presented as follows. Section 4.1 explains the sources of health data. Section 4.2.1 and Section 4.2.2 explore the results in detail and present a study of the forecasting power of the methods in a two-week horizon. Section 4.2.3 displays forecasts for the second wave. Section 4.2.4 aims to illustrate the robustness of the forecasting over longer periods of time. A discussion of the fitting errors of the methods is given in Appendix A. Additional results highlighting the accuracy of the forecasts are shown in Appendix B.

### 4.1. Data

**Figure 3:**
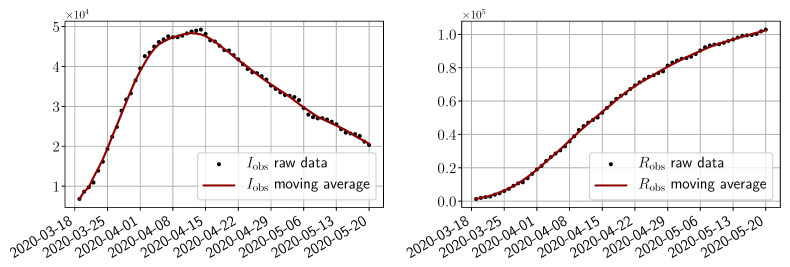
**Data from $t_{0}=19/03/2020$ to T=20/05/2020**.

We use public data from Santé Publique France (https://www.data.gouv.fr/en/datasets/donnees-hospitalieres-relatives-a-lepidemie-de-covid-19/) to get the numbers $I_{\mathrm{obs}}\left(t\right)$ of infected and $R_{\mathrm{obs}}\left(t\right)$ of removed people. As shown in Figure 3, the raw data present some oscillations at the scale of a week, which are due to administrative delays for the cases to be officially reported by hospitals. For our methodology, we have smoothed the data by applying a 7 day moving average filter. In order to account for the total number of infected people, we also multiply the data by an adjustment factor $f_{\mathrm{adj}}=15$ as stated in the literature (indeed, it is said in [29] that “of the 634 confirmed cases, a total of 306 and 328 were reported to be symptomatic and asymptomatic”, and in [10], it is claimed that the probability of developing severe symptoms for a symptomatic patient is 0 for children, 0.1 for adults and 0.2 for seniors; thus, if one takes p=0.13 as an approximate value of these probabilities, one may estimate the adjustment factor as $f_{\mathrm{adj}}=\frac{634}{328}\times \frac{1}{p}\approx 15$). Obviously, this factor is uncertain and could be improved in the light of further retrospective studies of the outbreak. However, note that when S≃N, which is the case at the start of the epidemic, the impact of this factor is negligible in the dynamics as can be understood from (3). In addition, since we use the same factor to provide a forecast of hospitalized people, the influence on the choice is minor.

### 4.2. Results

**Figure 4:**
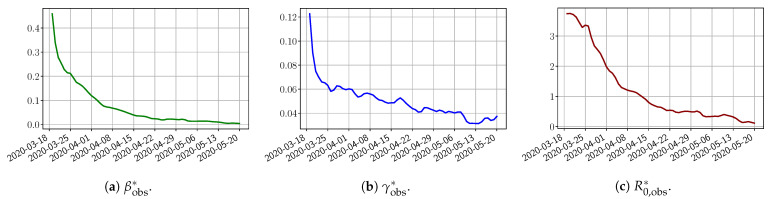
$\beta_{\mathrm{obs}}^{*}$, $\gamma_{\mathrm{obs}}^{*}$, $R_{0,\mathrm{obs}}^{*}=\beta_{\mathrm{obs}}^{*}/\gamma_{\mathrm{obs}}^{*}$ deduced from the data from $t_{0}=19/03/2020$ to T=20/05/2020

Using the observations $I_{\mathrm{obs}}\left(t\right)$ and $R_{\mathrm{obs}}\left(t\right)$, we apply a finite difference scheme in Formula (3) to derive $\beta_{\mathrm{obs}}^{*}\left(t\right)$ and $\gamma_{\mathrm{obs}}^{*}\left(t\right)$ for t∈[0,T]. Figure 4 shows the values of these parameters as well as the basic reproduction number $R_{0,\mathrm{obs}}^{*}=\beta_{\mathrm{obs}}^{*}/\gamma_{\mathrm{obs}}^{*}$ for the first pandemic wave in Paris.

We next follow the steps presented in Section 2.2 to obtain the forecasts. In the learning phase (step 1), we use two parametric detailed models of SE2IUR and SEI5CHRD types to generate training sets $B_{\mathrm{tr}}$ and $G_{\mathrm{tr}}$ composed of K=2618 training functions β(μ) and γ(μ) where μ are uniformly sampled in the set of parameters $P_{\mathrm{tr}}$ in the vicinity of the parameter values suggested in the literature [9,10]. Based on these training sets, we finish step 1 by building three types of reduced models: SVD, NMF and ENG (see Section 2.4).

Given the reduced bases $B_{n}$ and $G_{n}$, we next search for the optimal $\beta_{n}^{*}\in B_{n}$ and $\gamma_{n}^{*}\in G_{n}$ that best fit the observations (step 2 of our procedure). For this fitting step, we consider two loss functions:

1. routine-IR: loss function $J(\beta ,\gamma \;|\;I_{\mathrm{obs}},R_{\mathrm{obs}},\left[0,T\right])$ from (1),
2. routine-βγ: loss function $\tilde{J}\left(\beta ,\gamma \;|\;\beta_{\mathrm{obs}}^{*},\gamma_{\mathrm{obs}}^{*},\left[0,T\right]\right)$ from (6)

**Figure 5:**
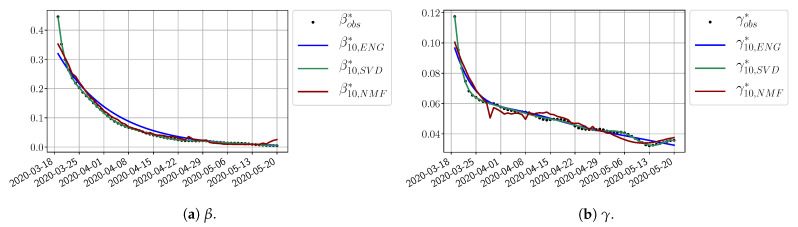
**Fitting from $t_{0}=19/03/2020$ to T=20/05/2020**.

We study the performance of each of the three reduced models and the impact of the dimension *n* of the reduced model in terms of the fitting error. The presentation of these results is presented in Appendix A in order not to overload the main discussion. The main conclusion is that the fitting strategy using SVD-reduced bases provides smaller errors than NMF and ENG, especially when we increase the number of modes *n*. This is illustrated in Figure 5 where we show the fittings obtained with routine-βγ and n=10 for the first wave (from $t_{0}=19/03/2020$ to T=20/05/2020). We observe that SVD is the best at fitting $\beta_{obs}^{*}$ and $\gamma_{obs}^{*}$, while ENG produces a smoother fitting of the data. Although the smoother fitting with ENG yields larger fitting errors than SVD, we see in the next section that it yields better forecasts.

#### 4.2.1. Forecasting for the First Pandemic Wave with a 14 Day Horizon

**Figure 6:**
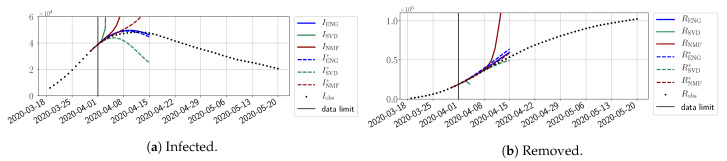
Fourteen-day forecasts starting from T=01/04.

**Figure 7:**
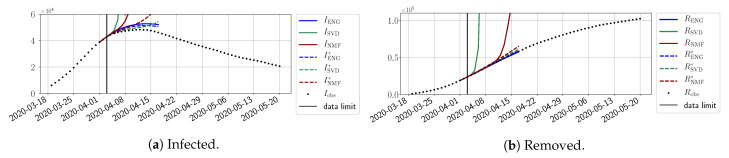
Fourteen-day forecasts starting from T=03/04.

**Figure 8:**
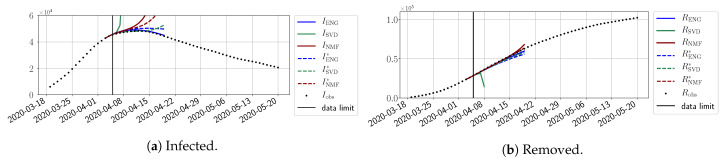
Fourteen-day forecasts starting from T=05/04.

**Figure 9:**
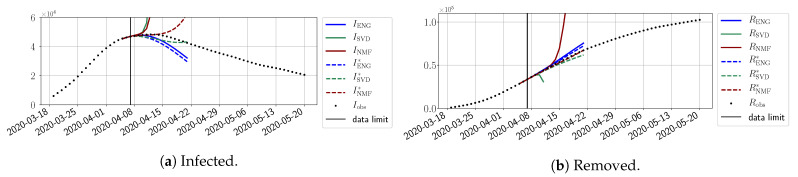
Fourteen-day forecasts starting from T=07/04.

**Figure 10:**
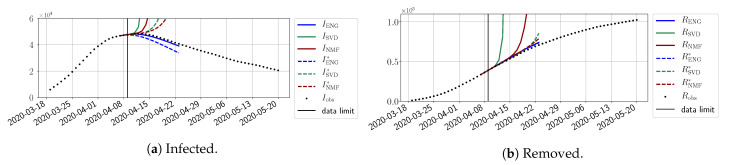
Fourteen-day forecasts starting from T=09/04.

**Figure 11:**
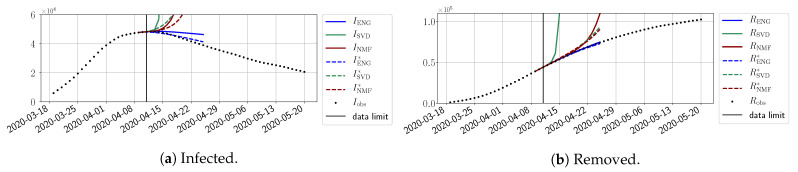
Fourteen-day forecasts starting from T=11/04.

**Figure 12:**
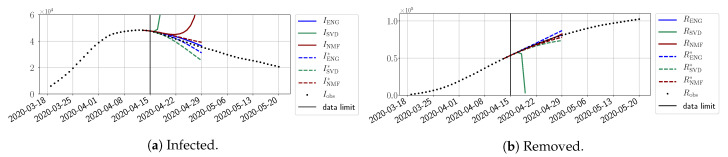
Fourteen-day forecasts starting from T=15/04.

**Figure 13:**
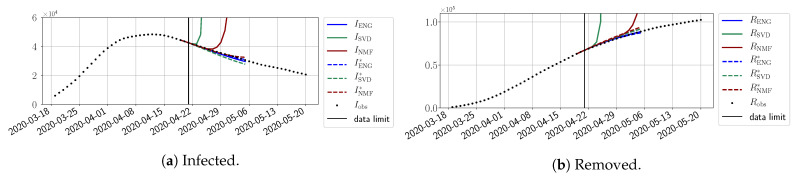
Fourteen-day forecasts starting from T=21/04.

**Figure 14:**
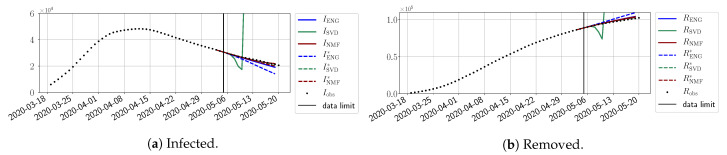
Fourteen-day forecasts starting from T=05/05.

In this section, we illustrate the short-term forecasting behavior of our method. We consider a forecasting window of τ=14 days and we examine several different starting days in the course of the first pandemic wave. The results are shown in Figure 6, Figure 7, Figure 8, Figure 9, Figure 10, Figure 11, Figure 12, Figure 13 and Figure 14. Recall that that the forecasting uses the coefficients of the reduced bases obtained by the fitting procedure but also the optimal initial condition of the forecast that minimizes the error on the three days prior to the start of the forecast. For each given fitting strategy (routine-IR, routine-βγ) and each given type of reduced model (SVD, NMF, ENG), we have chosen to plot an average forecast computed with predictions using reduced dimensions n∈{5,6,7,8,9,10}. This choice is a simple type of forecast combination, but of course other more elaborate aggregation options could be considered. The labels of the plots correspond to the following:

- $I_{SVD}$, $I_{NMF}$, $I_{ENG}$, $R_{SVD}$, $R_{NMF}$, $R_{ENG}$ are the average forecasts obtained using routine-βγ.
- $I_{SVD}^{*}$, $I_{NMF}^{*}$, $I_{ENG}^{*}$, $R_{SVD}^{*}$, $R_{NMF}^{*}$, $R_{ENG}^{*}$ are the average forecasts obtained using routine-IR.

The main observation from Figure 6, Figure 7, Figure 8, Figure 9, Figure 10, Figure 11, Figure 12, Figure 13 and Figure 14 is that the ENG-reduced model is the most robust and accurate forecasting method. Fitting ENG with routine-IR or routine-βγ does not seem to lead to large differences in the quality of the forecasts, but routine-βγ seems to provide slightly better results. This claim is further confirmed by the study of the numerical forecasting errors of the different methods shown in Appendix B.

Figure 6, Figure 7, Figure 8, Figure 9, Figure 10, Figure 11, Figure 12, Figure 13 and Figure 14 also show that the SVD-reduced model is very unstable and provides forecasts that blow up. This behavior illustrates the dangers of overfitting, namely that a method with high fitting power may present poor forecasting power due to instabilities. In the case of SVD, the instabilities stem from the fact that approximations are allowed to take negative values. This is the reason why NMF, which incorporates the nonnegative constraint, performs better than SVD. One of the reasons why ENG outperforms NMF is the enlargement of the cone of nonnegative functions (see Section 2.4). It is important to note that, with ENG, the reduced bases are directly related to well-chosen virtual scenarios, whereas SVD and NMF rely on matrix factorization techniques that provide purely artificial bases. This makes forecasts from ENG more realistic and therefore more reliable.

#### 4.2.2. Focus on the Forecasting with ENG

**Figure 15:**
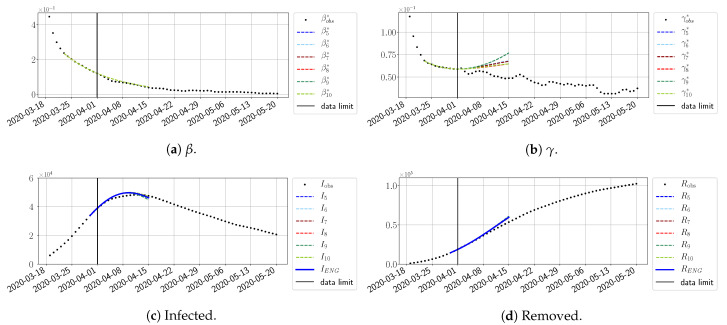
Enlarged Nonnegative Greedy (ENG) forecast from T=01/04.

**Figure 16:**
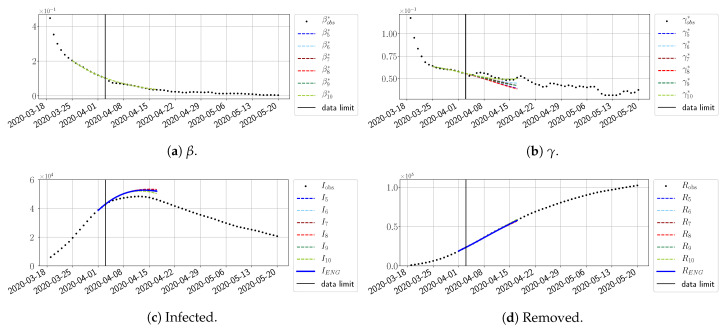
ENG forecast from T=03/04.

**Figure 17:**
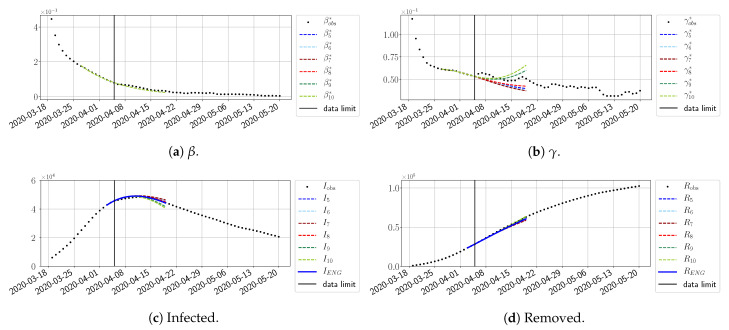
ENG forecast from T=05/04.

**Figure 18:**
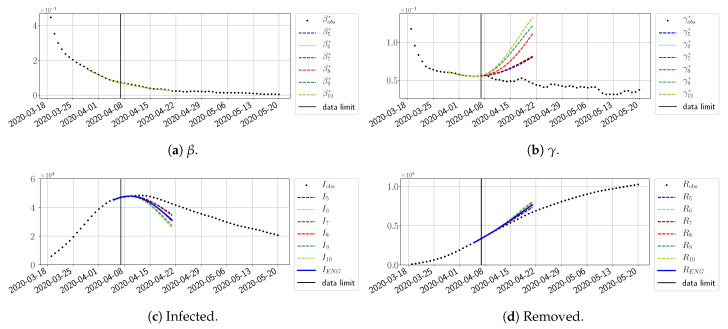
ENG forecast from T=07/04.

**Figure 19:**
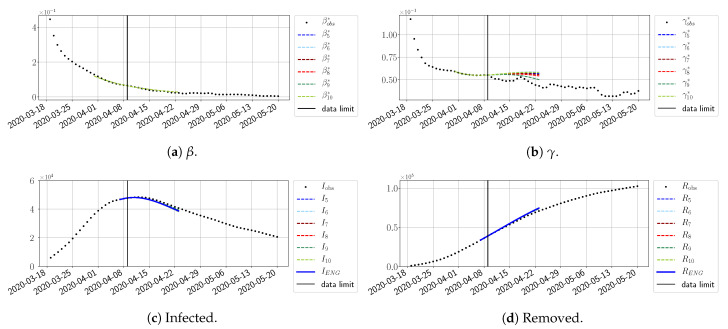
ENG forecast from T=09/04.

**Figure 20:**
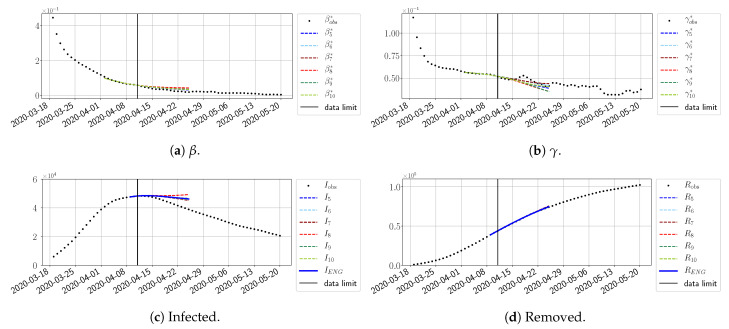
ENG forecast from T=11/04.

**Figure 21:**
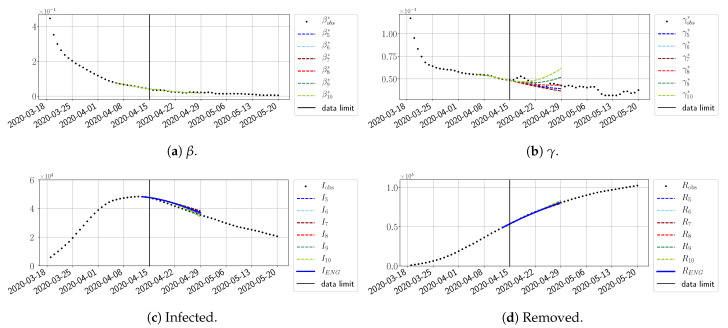
ENG forecast from T=15/04.

**Figure 22:**
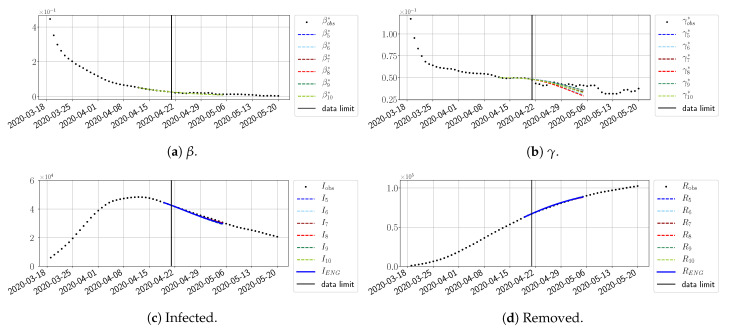
ENG forecast from T=21/04.

**Figure 23:**
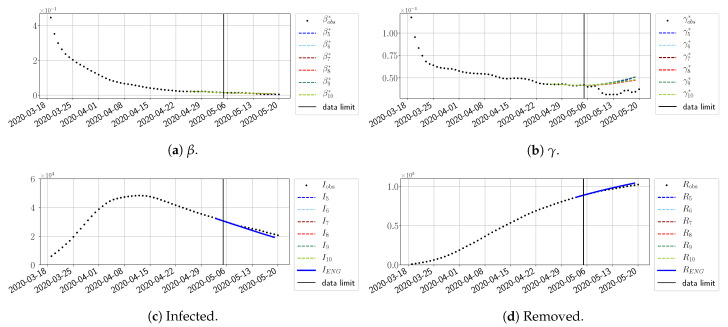
ENG forecast from T=05/05.

For our best forecasting method (routine-βγ using ENG), we plot in Figure 15, Figure 16, Figure 17, Figure 18, Figure 19, Figure 20, Figure 21, Figure 22 and Figure 23 the forecasts for each dimension n=5 to 10. The plots give the forecasts on a 14 day-ahead window for β, γ, and the resulting evolution of the infected *I* and removed *R*. We see that the method performs reasonably well for all values of *n*, proving that the results of the previous section with the averaged forecast are not compensating for spurious effects which could occur for certain values of *n*. We have chosen to display the inaccurate forecasts from 3 April, 7 April, and 11 April as they are among the worst predictions obtained using this method; however, it is important to mention that, despite the lack of accuracy in these cases, plausible epidemic behaviors remain, with different but realistic evolutions for β and γ compared to the actual evolution. Note that the method was able to predict the peak of the epidemic several days in advance (see Figure 15). We also observe that the prediction on γ is difficult at all times due to the fact that $\gamma_{\mathrm{obs}}^{*}$ presents an oscillatory behavior. Despite this difficulty, the resulting forecasts for *I* and *R* are very satisfactory in general.

#### 4.2.3. Forecasting of the Second Wave with ENG

**Figure 24:**
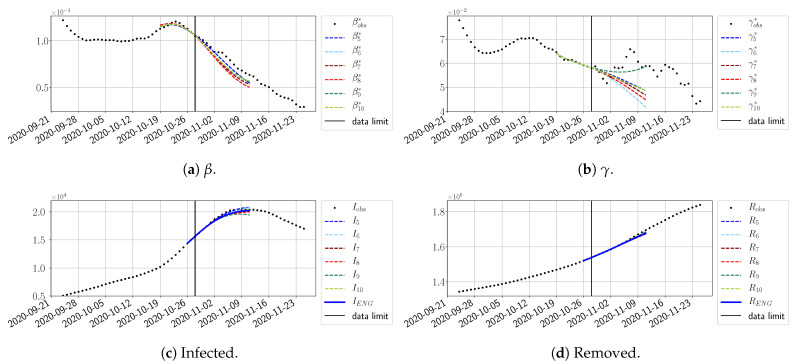
ENG forecast from T=28/10.

**Figure 25:**
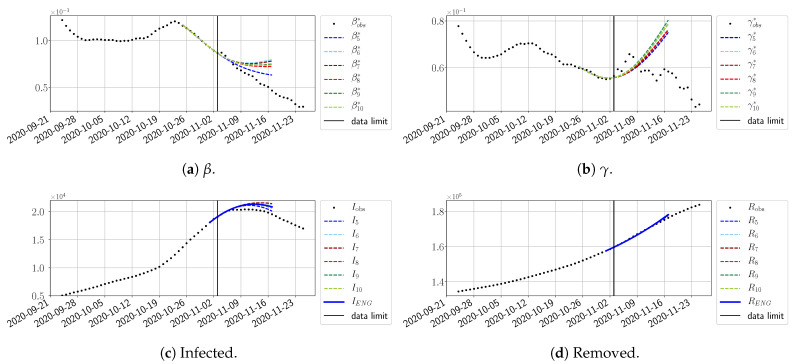
ENG forecast from T=03/11.

**Figure 26:**
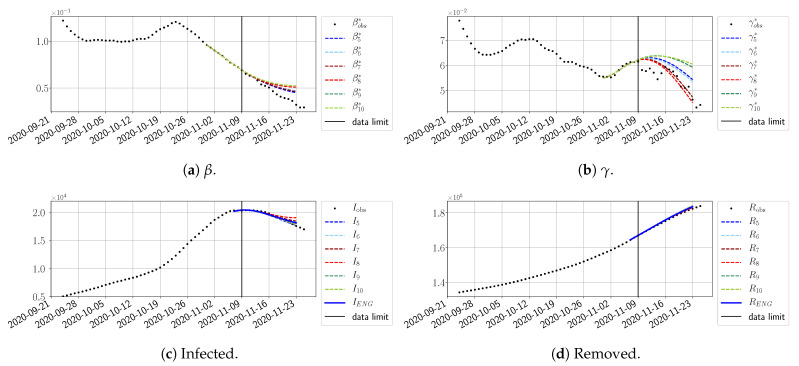
ENG forecast from T=09/11.

The review took place during the month of November 2020 as the second COVID-19 pandemic wave hit France. We took advantage of this to enlarge the body of numerical results, and we provide some example forecasts with ENG for this wave in Figure 24, Figure 25 and Figure 26. As the figures illustrate, the method provides very satisfactory forecasts in a 14 day-ahead window. We again observe a satisfactory prediction of the second peak (Figure 24, Figure 25 and Figure 26) and the same difficulty in forecasting γ due to the oscillations in $\gamma_{\mathrm{obs}}$, but this has not greatly impacted the quality of the forecasts for *I* and *R*.

#### 4.2.4. Forecasts with ENG with a 28 Day-Ahead Window

**Figure 27:**
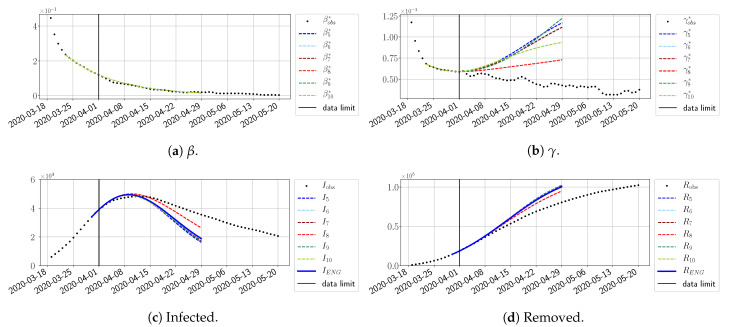
ENG forecast from T=01/04.

**Figure 28:**
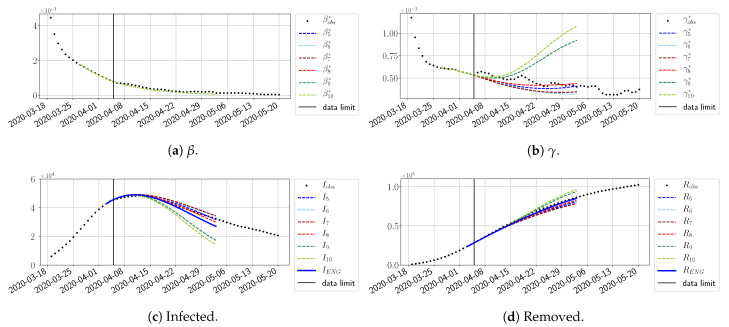
ENG forecast from T=05/04.

**Figure 29:**
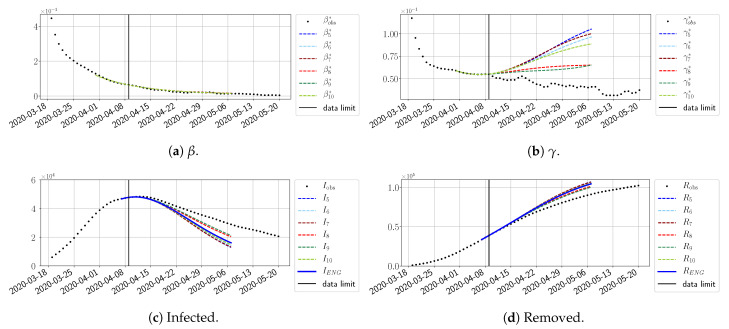
ENG forecast from T=05/04.

**Figure 30:**
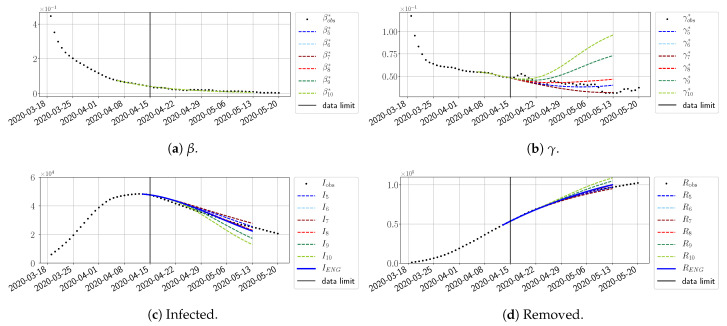
ENG forecast from T=15/04.

**Figure 31:**
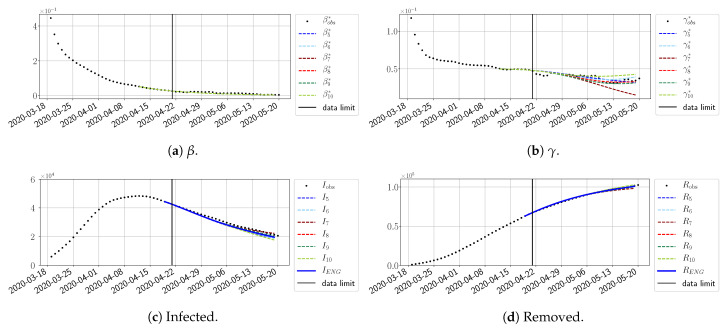
ENG forecast from T=21/04.

**Figure 32:**
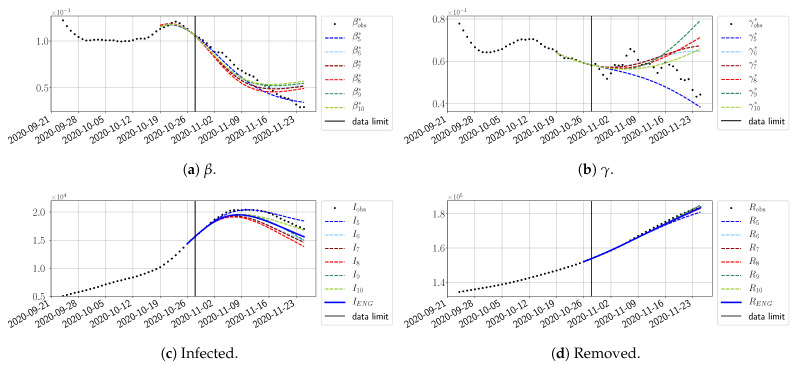
ENG forecast from T=28/10.

To conclude this section, we extend the forecasting window to 28 days instead of 14 and study whether the introduced ENG method still provides satisfactory forecasts. As shown in Figure 27, Figure 28, Figure 29, Figure 30, Figure 31 and Figure 32, the results of the methods are quite stable for large windows. This shows that, in contrast to standard extrapolation methods using classical linear or affine regressions, the reduced basis catches the dynamics of β and γ not only locally but also at extended time intervals.

## 5. Conclusions

We have developed an epidemiological forecasting method based on reduced modeling techniques. Of the different strategies that have been explored, the one that outperforms the rest in terms of robustness and forecasting power involves reduced models that are built with an Enlarged Nonnegative Greedy (ENG) strategy. This method is novel and of interest in itself as it allows reduced models that preserve positivity and even other types of bounds to be built. Despite the fact that ENG does not have optimal fitting properties (i.e., interpolation properties), it is well suited for forecasting since, due to the preservation of the natural constraints of the coefficients, it generates realistic dynamics with few modes. The results have been presented for a mono-regional test case, and we plan to extend the present methodology to a multi-regional setting using mobility data.

Last but not least, we would like to emphasize that the developed strategy is very general and could be applied to the forecasting of other types of dynamics. The specificity of each problem may, however, require adjustments in the reduced models. This is exemplified in the present problem through the construction of reduced models that preserve positivity.

## Figures and Tables

**Table 1:** Description of the compartments in Model SEI5CHRD.

Compartment	Description
*S*	Susceptible
*E*	Exposed (non infectious)
$I_{p}$	Infected and pre-symptomatic (already infectious)
$I_{a}$	Infected and a-symptomatic (but infectious)
$I_{ps}$	Infected and paucisymptomatic
$I_{ms}$	Infected with mild symptoms
$I_{ss}$	Infected with severe symptoms
*H*	Hospitalized
*C*	Intensive Care Unit
*R*	Removed
*D*	Dead

**Table 2:** Description of the parameters involved in Model SEI5CHRD.

Parameter	Description
$\beta_{p}$	Relative infectiousness of $I_{p}$
$\beta_{a}$	Relative infectiousness of $I_{a}$
$\beta_{ps}$	Relative infectiousness of $I_{ps}$
$\beta_{ms}$	Relative infectiousness of $I_{ms}$
$\beta_{ss}$	Relative infectiousness of $I_{ss}$
$\beta_{H}$	Relative infectiousness of $I_{H}$
$\beta_{C}$	Relative infectiousness of $I_{C}$
$\varepsilon^{-1}$	Latency period
$\mu_{p}^{-1}$	Duration of prodromal phase
$p_{a}$	Probability of being asymptomatic
$\mu^{-1}$	Infectious period of $I_{a}$, $I_{ps}$, $I_{ms}$, $I_{ss}$
$p_{ps}$	If symptomatic, probability of being paucisymptomatic
$p_{ms}$	If symptomatic, probability of developing mild symptoms
$p_{ss}$	If symptomatic, probability of developing severe symptoms (note that $p_{ps}+p_{ms}+p_{ss}=1$)
$p_{C}$	If severe symptoms, probability of going in C
$\lambda_{CR}$	If in C, daily rate entering in *R*
$\lambda_{CD}$	If in C, daily rate entering in *D*
$\lambda_{HR}$	If hospitalized, daily rate entering in *R*
$\lambda_{HD}$	If hospitalized, daily rate entering in *D*

**Table 3:** Description of the compartments in model SE2IUR.

Compartment	Description
*S*	Susceptible
$E_{1}$	Exposed (non infectious)
$E_{2}$	Infected and pre-symptomatic (already infectious)
*I*	Infected and symptomatic
*U*	Un-noticed
*R*	dead and removed

**Table 4:** Description of the parameters involved in model SE2IUR.

Parameter	Description
β	Relative infectiousness of *I*, *U*, $E_{2}$
$\delta^{-1}$	Latency period
$\sigma^{-1}$	Duration of prodromal phase
ν	Proportion of *I* among I+U
$\gamma_{1}$	If *I*, daily rate entering in *R*
$\gamma_{2}$	If *U*, daily rate entering in *R*

**Figure A1:**
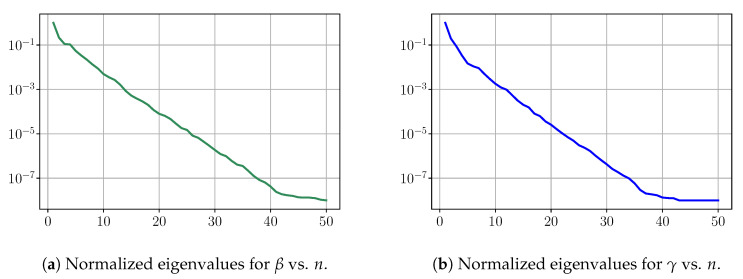
Training step: Decay of SVD eigenvalues.

**Figure A2:**
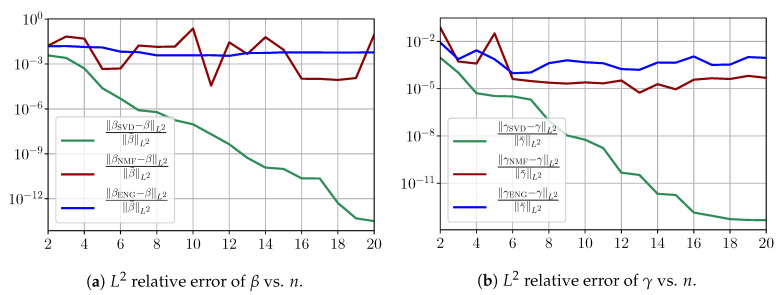
Study of sampling errors: Fitting errors of functions β and γ from B and G that belong to the training sets $B_{\mathrm{tr}}$ and $G_{\mathrm{tr}}$.

**Figure A3:**
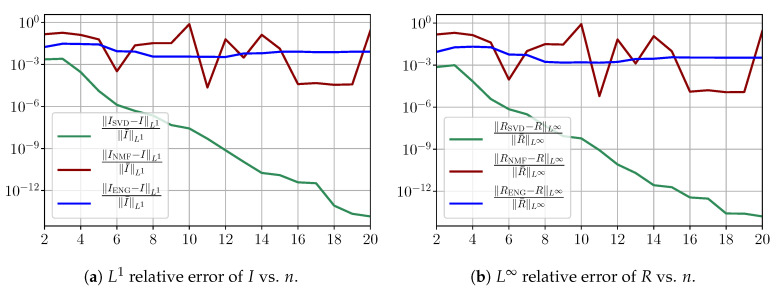
Study of sampling errors: Errors on I and R obtained by the susceptible–infected–removed (SIR) model using the fitted β and γ.

**Figure A4:**
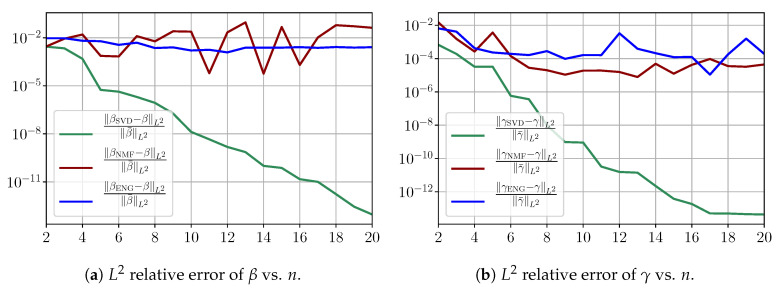
Study of sampling errors: Fitting errors of functions β and γ from B and G but which do not belong to the training sets $B_{\mathrm{tr}}$ and $G_{\mathrm{tr}}$.

**Figure A5:**
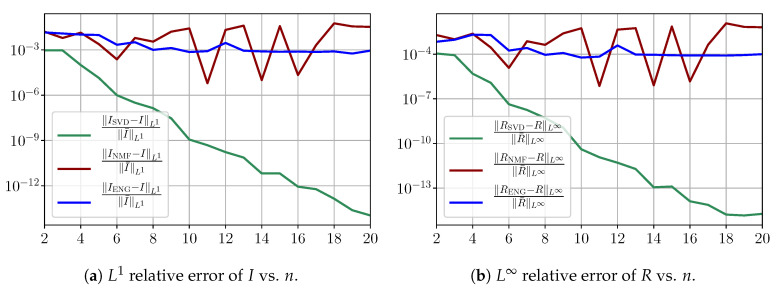
Study of sampling errors: Errors on I and R obtained by the SIR model using the fitted β and γ.

**Figure A6:**
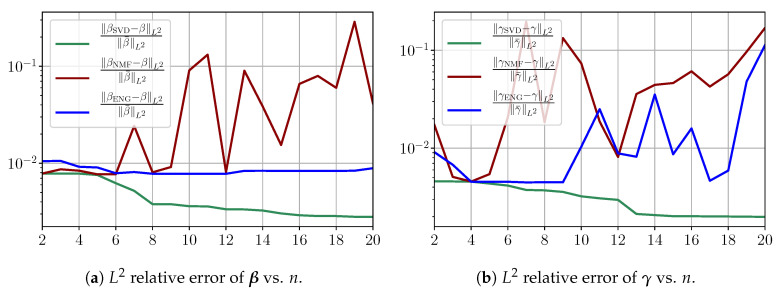
Fitting errors of β and γ in a noisy virtual scenario.

**Figure A7:**
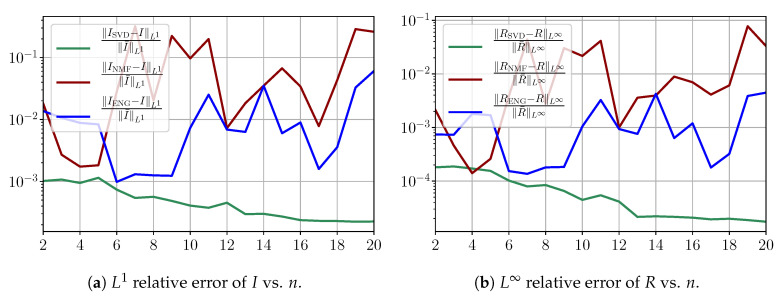
Fitting errors of *I* and *R* on noisy data.

**Figure A8:**
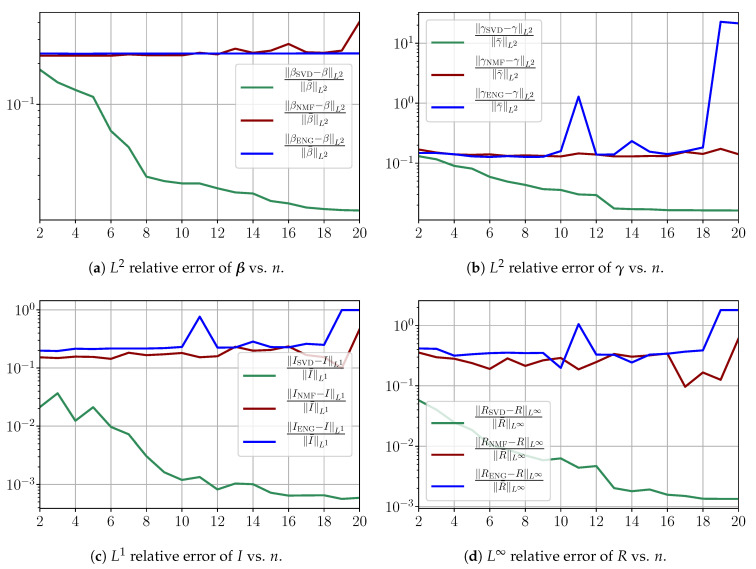
**Fitting errors from $t_{0}=19/03/2020$ to T=03/05/2020**.

**Figure A9:**
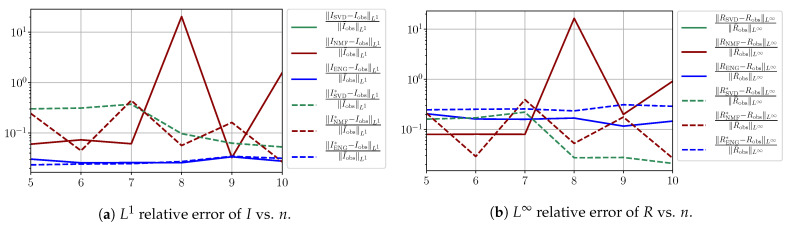
Forecasting errors of *I* and *R* (from T=01/04).

**Figure A10:**
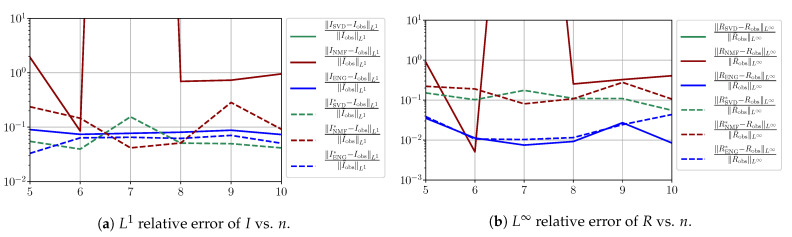
Forecasting errors of *I* and *R* (from T=03/04).

**Figure A11:**
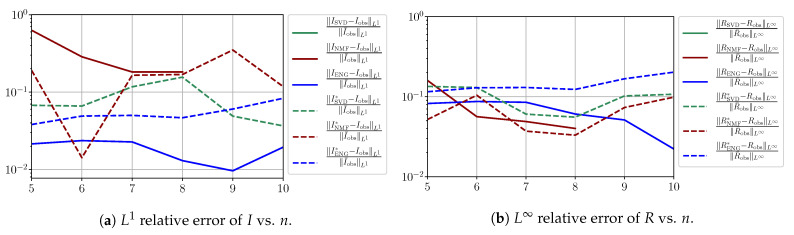
Forecasting errors of *I* and *R* (from T=05/04).

**Figure A12:**
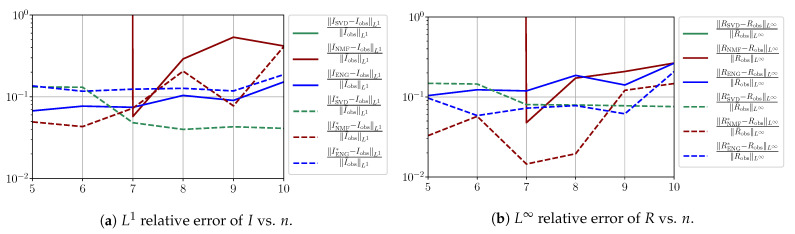
Forecasting errors of *I* and *R* (from T=07/04).

**Figure A13:**
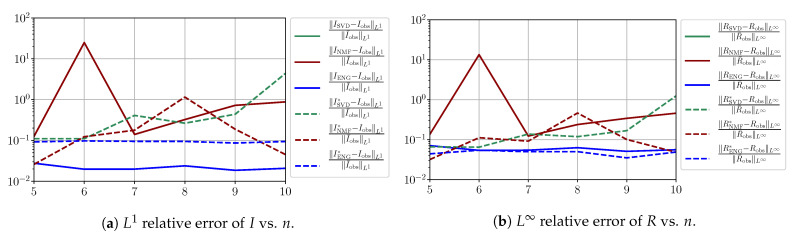
Forecasting errors of *I* and *R* (from T=09/04).

**Figure A14:**
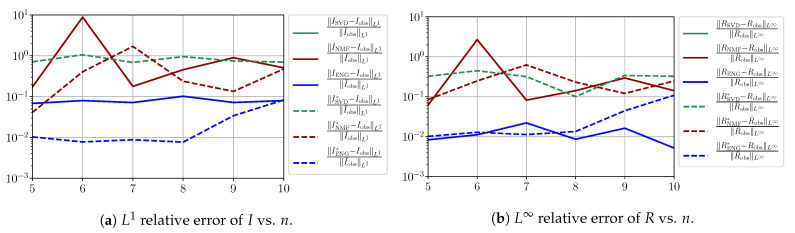
Forecasting errors of *I* and *R* (from T=11/04).

**Figure A15:**
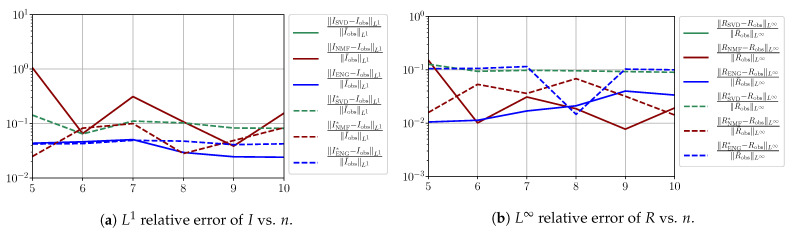
Forecasting errors of *I* and *R* (from T=15/04).

**Figure A16:**
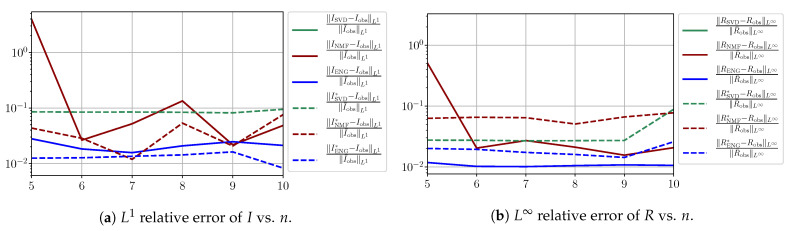
Forecasting errors of *I* and *R* (from T=21/04).

**Figure A17:**
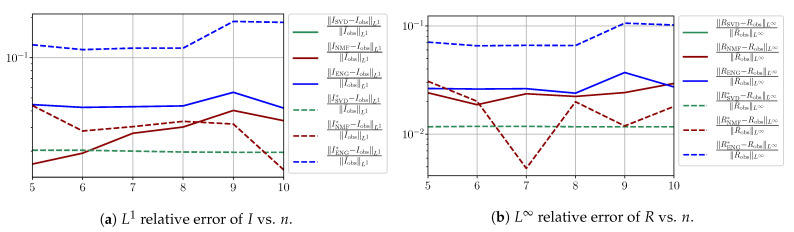
Forecasting errors of *I* and *R* (from T=05/05).

## Data Availability

Data available on request.

## Appendix A. Analysis of Model Error and Observation Noise

In this section, we study the impact of observation noise and model error on the quality and behavior of the fitting step. The elements that impact the accuracy of our procedure are the following:

1. Observation noise: The observed health data $I_{\mathrm{obs}}$ and $R_{\mathrm{obs}}$ present several sources of noise. There may be some inaccuracies in the reporting of cases, and the data are then post-processed. This eventually yields the noisy time series $I_{\mathrm{obs}}$ and $R_{\mathrm{obs}}$ for which it is difficult to estimate the uncertainty. These noisy data are then used to produce (through finite differences) $\beta_{\mathrm{obs}}^{*}$ and $\gamma_{\mathrm{obs}}^{*}$, which are in turn also noisy.
2. Model errors: Two types of model errors are identified:
  (a) Intrinsic model error on B and G: The families of detailed models that we use are rich, but they may not cover all possible evolutions of $I_{\mathrm{obs}}$ and $R_{\mathrm{obs}}$. In other words, our manifolds B and G may not perfectly cover the real epidemiological evolution. Thsi error motivated the introduction of the exponential functions $b_{0}$ and $g_{0}$ described in Section 2.4.
  (b) Sampling error of B and G: The size of the training sets $B_{\mathrm{tr}}$ and $G_{\mathrm{tr}}$ and the dimension *n* of the reduced models $B_{n}$ and $G_{n}$ also limit the approximation capabilities of the continuous target sets B and G.

In this section, we aim to disentangle these effects in order to give a better insight of the fitting error behavior for the real data.

### Appendix A.1. Study of the Impact of the Sampling Error: Fitting a Virtual Scenario

Our set of virtual epidemiological dynamics is U. After the collapsing step, the manifolds for β and γ are B and G. These sets are potentially infinite, and we have used finite training subsets $B_{\mathrm{tr}}\subseteq B$ and $G_{\mathrm{tr}}\subseteq G$ to build the reduced models $B_{n}$ and $G_{n}$.

First, we consider the eigenvalues for β and γ when performing an SVD decomposition for the virtual scenarios. Figure A1 shows a rapid decay of the eigenvalues obtained by SVD decomposition and shows that we can obtain a very good approximation of elements of $B_{\mathrm{tr}}\subseteq B$ and $G_{\mathrm{tr}}\subseteq G$ with only a few modes.

Among the sources of noise and model error given at the beginning of Appendix A, we can study the impact of the sampling error (point 2-b) as follows. For this, we start by examining the fitting error on an interval of 45 days for two functions β and γ which belong to the manifold B and G and are in the training sets $B_{\mathrm{tr}}$ and $G_{\mathrm{tr}}$. This error will serve as a benchmark, which we use to compare the fitting errors of functions that are not in the training sets.

Figure A2 shows relative fitting errors $∥\beta_{n}^{*}-\beta_{\mathrm{obs}}^{*}{∥}_{L^{2}\left(\left[t_{0},T\right]\right)}/{∥{\bar{\beta }}_{\mathrm{obs}}^{*}∥}_{L^{2}\left(\left[t_{0},T\right]\right)}$ and $∥\gamma_{n}^{*}-\gamma_{\mathrm{obs}}^{*}{∥}_{L^{2}\left(\left[t_{0},T\right]\right)}/{∥{\bar{\gamma }}_{\mathrm{obs}}^{*}∥}_{L^{2}\left(\left[t_{0},T\right]\right)}$ using SVD, NMF and ENG-reduced bases with n=2,…,20, where the notation $\bar{x}$ denotes the mean of the quantity *x* over [0,T]. We observe that, for SVD, the fitting errors behave similarly to the error decay of the training step (Figure A1).

Figure A3 shows the $L^{1}$ and $L^{\infty }$ errors obtained after the propagation of the fittings of β and γ. In both metrics, the error for I and R obtained using SVD decreases below $10^{-12}$. When NMF and ENG are used, the error decreases and stagnates at $10^{-2}$ for both I and R.

Now, we consider the fitting error for two functions β and γ which belong to the manifold B and G but which are not in the training sets $B_{\mathrm{tr}}$ and $G_{\mathrm{tr}}$. Figure A4 shows the fitting error on the virtual scenario considered using SVD, NMF and ENG-reduced bases for n=2,…,20. We note that the quality of the fittings in each method is very similar to that of Figure A3 where the functions were taken in the training sets. This illustrates that the sampling error does not play a major role in our experiments.

Figure A5 shows the $L^{1}$ and $L^{\infty }$ errors obtained after the propagation of the fittings of β and γ. In both metrics, the error for I and R obtained using SVD decreases to $10^{-14}$. When NMF and ENG are used, the error decreases and stagnates at $10^{-3}$ and $10^{-4}$ for both I and R, respectively.

### Appendix A.2. Study of the Impact of Noisy Data and Intrinsic Model Error

To investigate the impact of noise in the observed data, we now add noise to the two previously chosen functions β∈B and γ∈G, and we study the fitting error for this noisy data. The level of the noise has been chosen to be the same level as that estimated for the real dynamics. In order to estimate the noise, we performed a fitting of the real data using SVD-reduced bases; the level of noise is defined as the difference between this fitting and the real data. This level of noise is then added to the virtual scenario considered here. Figure A6 shows the fitting error on β and γ using SVD, NMF and ENG-reduced bases for n=2,…,20.

We observe that the noise strongly deteriorates the fitting error obtained using NMF, and the error becomes oscillatory and very unstable. For ENG, the error remains low and consistently around $10^{-2}$ for β. We observe the same behavior for γ with instabilities arising for n>10. For SVD, the error is lower than in the ENG case and slowly decreases as *n* increases. Figure A7 shows the $L^{1}$ and $L^{\infty }$ errors obtained after the propagation of the fittings of β and γ. In line with the observations from Figure A6, the error obtained using NMF is very unstable. Using ENG, we observe a decay from n=2 to n=7 and oscillations that remain around $10^{-2}$ for *I* and $10^{-3}$ for *R*. The decay observed for SVD is slow and steady; the error nearly reaches $10^{-4}$ for *I* and $10^{-5}$ for *R* when n=20.

Finally, it is necessary to add the intrinsic model error on top of the previous sampling error and observation noise. In so doing, the main change is that the previous fitting error plots from Figure A6 have essentially the same behavior but the error values are increased depending on the degree of model inaccuracy. We have therefore disentangled all the effects of model error and noisy data, and all the observations from this section thus give a better insight regarding the fitting on the real data.

Figure A8 summarizes the fitting results for an example fitting period taken from $t_{0}=19/03$ to T=03/05. Figure A8a,b shows the fitting error on β and γ using SVD, NMF and ENG-reduced bases for n=2,…,20. Figure A8c,d shows the $L^{1}$ and $L^{\infty }$ relative errors on $I_{n}$ and $R_{n}$ after the propagation of the fittings of $\beta_{n}^{*}$ and $\gamma_{n}^{*}$.

From Figure A8a,b, we observe that the fitting error with SVD decreases at a moderate rate as the dimension *n* of the reduced basis is increased. The error with NMF and ENG does not decrease and oscillates around a certain constant error value of the order $10^{-1}$. Note that this value is small and yields small errors in the approximation of I and R, as Figure A8c,d illustrates.

## Appendix B. Study of Forecasting Errors

In this section, we present a detailed study of the forecasting errors for each different fitting strategy (routine-IR, routine-βγ), reduced model (SVD, NMF, ENG) and starting date *T*. We anticipate the main conclusion announced in Section 4.2.1: ENG fitted with routine-βγ outperforms the other reduced models and is the most robust and accurate reduced model to use in a forecasting strategy. Figure A9 shows the relative errors of a 14 day forecast from T=01/04 for each forecasting method and each reduced basis. Similarly Figure A10,Figure A11,Figure A12,Figure A13,Figure A14,Figure A15,Figure A16,Figure A17 show the forecasts’ relative errors from different times, respectively.

We observe that the quality of the forecast depends on the reduced basis but also strongly on the starting day *T* from which the forecast is produced. The forecasts using routine-βγ with SVD and NMF are not accurate and most often blow up. When routine-IR is used with SVD and NMF, the forecasts are more robust as there is no observed explosion of error. Reduced bases from ENG consistently lead to the the best forecast being obtained using either routine-βγ and routine-IR; by inspecting the error on Figure A9,Figure A10,Figure A11,Figure A12,Figure A13,Figure A14,Figure A15,Figure A16,Figure A17 and the averaged forecasts obtained in Section 4.2.1, we conclude that routine-βγ with ENG-reduced bases provides slightly better forecasts.
